# Populist ideology, ideological attitudes, and anti-immigration attitudes as an integrated system of beliefs

**DOI:** 10.1371/journal.pone.0280285

**Published:** 2023-01-17

**Authors:** Valerio Pellegrini

**Affiliations:** Department of Social and Developmental Psychology, Sapienza University, Rome, Italy; University of Massachusetts, UNITED STATES

## Abstract

A challenge for the identification of the core components of a beliefs system is the topological examination of these components within the overall structure of the said system. By modeling beliefs as nodes of interconnected networks, this research investigated the centrality of adherence to populist ideology and classical ideological attitudes in relation to voting behavior and negative feelings toward immigrants. Data from a sample of 774 Italian adults were examined by means of three *Network Analysis* models. Results showed four constitutive dimensions of populist ideology: People Sovereignty, Anti-elitism, People Homogeneity, and Manichaeism. The dimensions of Anti-elitism, People Sovereignty and Homogeneity were found to be the core. Analyses also highlighted the centrality of *right-wing authoritarianism* (RWA) and *social dominance orientation* (SDO) within the broader beliefs system, including voting, populist ideology dimensions, and anti-immigration. RWA was positively related to the core of populist ideology, whilst SDO was negatively associated with or unrelated to it. However, both RWA and SDO exceeded populist dimensions when associated with populist right-wing voting, representing the unique intermediate links in connecting it with anti-immigration. Five Star Movement voting emerged as a purer form of populist support, relating directly only to populist dimensions and placing itself at a greater distance from ideological attitudes and anti-immigration.

## Introduction

During the last decade, populist parties and untraditional movements of different political leanings have risen to international prominence [1]. Responses from traditional parties and ideologies to the rise of populism have been often ineffectual or counterproductive. Hilary Clinton’s “basket of deplorables” definition was certainly not successful, to put it mildly. Responses to populism have often been of the technocratic type, arguably enhancing the feeling of political alienation that fueled the rise of populism in the West [2, 3].

Most attempts at opposing populism politically have been hampered by populism’s remarkable ideological flexibility. Such flexibility has allowed populism to intermingle with traditional left- or right-leaning policies and ideologies, and it has led some scholars to define populism as a fuzzy concept [4], or a thin-centered ideology [5].

Albeit thin-centered or fuzzy, populism articulates specific themes or narratives. Three main narratives are used to frame almost any possible policy: the stark opposition of two internally homogeneous groups—the “people” and the “elite”; a Manichean view of these groups (elite = evil); the view that politics should express the “General Will” of the people [5]. These narratives have been operationalized as constitutive dimensions of populist ideology and used to develop several scales designed to measure voters’ adherence to such ideology [e.g., 6–8]. Nevertheless, a clear and consensual view of the centrality of each component characterizing populism has not been attained. Thus, the present research first aimed to fill this gap by investigating the extent of the centrality of distinct components of populist ideology within populism’s interconnected network of narratives.

A further consequence of populist ideology’s thin-centered quality is the apparent need to borrow themes and issues belonging to classical “thick” ideologies [5]. Populist parties may flavor their narratives by drawing from exclusionary nativism, or from egalitarian and socialist tenets. For instance, in Spain and Greece, populist parties (Podemos and Syriza, respectively) with a strong electoral following rely on themes reminiscent of a socialist ideology, such as support for social equality and wealth redistribution. Thus, these parties are instantiations of left-wing leaning populism. However, the Italian Lega (League), the French Rassemblement National (known as the National Front until 2018), and the Spanish Vox, among other similar European parties, have intercepted a relevant segment of the electorate focusing on nativism, and by opposing immigration and diversity. Such parties embody the right-wing strain of populism [9]. Populism’s ability to assume the semblance of more tightly structured ideologies suggests that its success and the appeal of its narratives might be linked to psychological factors that are key for endorsing classical ideologies, such as authoritarianism and egalitarianism [10–12]. Thus, the present research also aimed to explore how classical ideological attitudes indexed by right-wing authoritarianism [RWA; 13] and social dominance orientation [SDO; 14] relate within a network including populism’s main narratives. SDO and RWA may hold together the different narratives typical of the populist discourse and link them to voting behavior and to a key policy preference—anti-immigration attitudes.

## Operationalization of the adherence to populist ideology

Although populism represents a concept of central relevance in the current landscape of social sciences, it remains a substantially contested concept in terms of its antecedents, consequences, and operationalizations [15]. A recent line of research has investigated populism at the individual level [e.g., 6] under the assumption that populist ideas should resonate with those of voters to be influential [16]. The premise of this line of research is that understanding populism at the individual level may help the interpretation of its diffusion, and how it may impact pluralist democratic systems [17]. From this line of research, several operationalizations of populist ideology have been provided, which have focused on a specific set of attitudes that embody its key narratives. Arguably, the most widely used has been provided by Akkerman et al. [6]. It synthesized the populist fuzzy ideology as a one-dimensional construct, trying to tap into different belief domains: belief in popular sovereignty entailing a negative view of representative government; the notion of people having more in common with each other than with the elite; and a black/white Manichean polarity between the pure people and the corrupted elite [6, 18]. However, it has been argued that the one-dimensional nature of the measure limits its ability to render a comprehensive picture of the facets of the populist fuzzy ideology [e.g., 19]. Relying on work by Wirth et al. [8], Schulz et al. [19] developed a three-dimensional inventory to represent populism: “Anti-elitism attitudes,” “Demand for people’s sovereignty,” and “Belief in a homogeneous and virtuous people.” In a similar multidimensional vein, Castanho Silva et al. [7] proposed a further three-dimensional measure. Castanho Silva et al. [7] borrowed two concepts from Schulz et al. [19]—“Anti-elitism attitudes” and “Demand for people’s sovereignty”—and replaced “People’s homogeneity” [19] with a “Manichean outlook” dimension.

Comparing these scales, a slight inconsistency among the proposed operationalizations of populist ideology emerges (see Table 1). Wuttke et al. [17] underlined that the unambiguous consensus of the different operative translations of populism lies in the recognition of the construct’s multidimensional nature. However, the dimensions representing its contents are less univocal across scholars. This ambiguity lies mainly in neglecting the conceptual complexity proposed by populist theorists and in the difficulty of translating the outlined conceptual characteristics into effective operationalizations [17].

**Table 1:** Comparative summary of the three measures of adherence to populist ideology.

Measure	N. Dimensions	N. Items	Dimensions and Reliability					
			Dimension 1	α	Dimension 2	α	Dimension 3	α
Akkerman et al., 2014	1	6	Populist Attitudes	.80				
Wirth et al., 2016/ Schulz et al., 2018	3	12	Anti-elitism Attitudes	.72	Demand for People’s Sovereignty	.81	Belief in a Homogeneous and Virtuous People	.78
Castanho Silva et al., 2018	3	9	Anti-elitism	.67	People-centrism	.47	Manichean Outlook	.31

*Note*. The Akkerman et al. (2014)’ measure, though one-dimensional, aims to detect: belief in popular sovereignty with a negative view of elite; the notion of people having more in common with each other than with the elite; black/white Manichean polarity of people and elite.

## Variants of populism across Europe

The systematization of populist narratives operated by the works described above has enabled its theoretical characteristics to be outlined. It offered a general frame of the beliefs range that shape populism’s identity as a thin-centered ideology [9]. The question of how populism’s themes connect with traditional ideologies, however, remains challenging because of the said fuzzy ideological nature of populism. To tackle the problem, researchers have investigated how the different parties in different countries embodied right- or left-leaning varieties of populism [e.g., 20]. Zulianello [21] summarized 66 populist parties from 33 European countries into three main macro-categories. The first two macro-categories of parties followed the classic definitions of right-wing and left-wing populism, understood in terms of a relative propensity for egalitarianism [22]. Right-wing populism, also referred to as exclusionary populism, adopts exclusionist narratives based on forms of chauvinism aimed at protecting the native citizens neglected by welfare and purportedly threatened by immigrants, supporting radical reforms aimed at depriving immigrants of civil rights, and providing for the definition of People by drawing on forms of cultural discrimination [23]. Examples of European political parties that could be included in this definition of populism were the Danish People’s Party in Denmark, Alternative for Germany in Germany, Vox in Spain, Party for Freedom in the Netherlands, and—in Italy—the League, Brothers of Italy (Fratelli d’Italia), and Forza Italia [21]. On the other hand, left-wing populism—or inclusionary populism—shifts the narrative level to positions close to socialist tenets. It proposes a redistribution of wealth, forms of political mobilization, such as direct democracy bypassing representative channels, and giving back voice and dignity to “forgotten” citizens. In Europe, there are several examples of populist parties that meet the ideological definition of the left-wing, such as Podemos in Spain, La France Insoumise in France, and Syriza in Greece.

In Italy, the left-wing variant of populism is not clearly embodied by any party of national relevance. The only political entity that sometimes, and rather ambiguously, seems to assume this semblance is represented by the Five Star Movement (M5S), a party that won a plurality in the general election of 2018, with more than 30% of the vote. However, as Mosca and Tronconi [24] have pointed out, it is difficult to meaningfully place this party along the left–right continuum. In a comparative study of the main European inclusionary populist parties, Font et al. [23] found that the M5S fell in the middle of the exclusionary and inclusionary poles, being clearly distinct from the two parties of SYRIZA and Podemos (pure instantiations of an inclusionary narrative). In the mapping work of Zulianello [21], the M5S, together with other similar phenomena (e.g., GERB in Bulgaria, MOST in Croatia, and ANO 2011 in the Czech Republic), is placed in a third innovative category defined as *valence populism*. Valence populism focuses on supposedly non-ideological issues, such as the fight against corruption, greater transparency, democratic reform, and moral integrity, while emphasizing anti-establishment motives. The distinctive feature of valence populists is precisely the prevailing emphasis on non-ideological issues, such as competence or honesty [21]. The parties adhering to this variety of populism seem to represent its quintessence as a thin-centered ideology, free from classical ideological markers, which is elastic, fluctuating, and sometimes contradictory.

## Thick-centered ideology and the thin-centered populism

This overview of European populist parties has highlighted the ability of populism to assume different ideological shapes. However, such a description does not say much about the psychological factors that successfully allow the transition of populism from “thin” to “thick” ideology that may occur when populist movements turn hard to the left, or to the right [9]. Unlike traditional ideologies, populism presents essential ideas or beliefs that are not sufficiently buttressed by a structured ideology and that can be applied to a wide range of political issues, but which lack the ability to be translated into a consistent policy position [5]. Therefore, it needs to adopt concepts from thicker ideologies, such as liberalism, socialism, or nativism. The malleability of populist ideology suggests that support for it, and the assumption of certain stances on various social issues, might be explained by resorting to psychological proclivities and motives that are known as buttressing the endorsement of classical ideologies, as well as their policy implications (e.g., anti-immigration stances and redistribution of wealth). The motivational goals concerning social change or social inequality can represent key psychological dimensions in conveying these dynamics [e.g., 10–12]. RWA and SDO are the sociopolitical attitudes most attuned to the resistance to change and acceptance of inequality [e.g., 25–28]. As such, RWA and SDO underpin left/right ideological preferences [e.g., 26, 29], as well as more specific attitudes—for instance, prejudice [e.g., 30, 31].

RWA taps the tendency to submit to the authorities, to adhere to the conventions and norms of society, and to endorse hostile attitudes toward those who do not adhere to them [13]. Thus, authoritarianism has been related to conservative attitudes and support for right-wing parties, as well as to prejudices toward minority groups [13, 26, 32]. SDO pertains to the adherence to an ideological attitude favorable to a hierarchical structure of society and reflects the desire for ingroup superiority compared to the outgroup [14]. Hence, SDO is associated with prejudice against ethnic minorities, as well as other low-status groups [26].

RWA and SDO place the emphasis on issues that seem to be in line with populist themes, particularly those of right-wing populism [12]. RWA is consistent with populist narratives defining the boundaries of the People through cultural discrimination. SDO, on the other hand, fits with exclusionary narratives based on forms of welfare chauvinism. As for valence populism, RWA and SDO might assume relevance contingent on the salience of themes in the specific political context. Valence populist parties (such as the Italian M5S) may fluctuate between different narratives in a contradictory way. If a relevant issue is in line with an exclusionary view (e.g., anti-immigration issues), they may veer toward adopting the stance instrumentally in order to nurture consensus. Thus, valence populism might find it convenient to harness the power inherent in the underlying motives of RWA and SDO.

## Research overview and hypotheses

The range of beliefs that shape populism’s identity as a thin-centered ideology may be summarized in four main narratives: Anti-elitism, People Sovereignty, People Homogeneity, Manichaeism [6–8, 19]. However, the available measures trudge to simultaneously capture these conceptual characteristics of populism and, at times, to translate them into effective operative definitions [17]. The present research aims to probe the structure of the adherence to populist ideology by combining the manifold beliefs inherent the different measures, with the exploratory purpose of assessing whether they effectively reflect the above-mentioned conceptual features.

Moreover, a clear and consensual view has not been attained of how central or pivotal each component characterizing populism is. The beliefs included in the abovementioned scales have not been investigated topologically in terms of the centrality of each belief within an ideological network, however fuzzy. This research aimed to fill this gap and to investigate to what extent distinct components of populist ideology are central within an interconnected network of narratives. Such a network of interconnected beliefs was investigated with the purely descriptive purpose of highlighting its core, central, and peripheral components.

Describing how central each belief or narrative is in the network helps to disambiguate the specific pull of the distinct dimensions of the populist attitudes in populism’s space. However, this does not highlight the psychological underpinnings of populism’s transition from a thin to a thick ideology. The thin-centered nature of populism means that it needs other additional ideological elements that are crucial to convey political meaning to voters [9]. Populist parties strive to broaden their support base by pandering to the concerns of public opinion, and by positioning themselves on the issues that define political discourse within the polity [33]. In Italy, during the period preceding the General election of 2018, two main populist parties dominated the political backdrop: the M5S and the League. The latter was part of a broader populist right-wing coalition, including Brothers of Italy and Forza Italia. Both these distinct populist entities aimed to gain an electoral consensus in specific constituencies. At the time, a key issue that topped voters’ concerns was immigration [see for example, 34, 35]. Political parties strove to position themselves on the issue. The League adopted a nativist understanding of the People, which was embedded in an exclusionary nationalist ideology [36]. In order not to be outflanked by its right-wing populist competitor, the M5S incrementally moved to more negative stances on immigration [37]. In this context, constructs defining right-wing orientation and anti-immigration attitudes, such as SDO and RWA, could represent pivots around which populist voting, beliefs, and its policy correlates may gravitate. In other words, RWA and SDO are expected to be key variables in linking populist narratives, populist voting, and anti-immigration attitudes.

## Method

### Participants

In exchange for course credit, psychology students were asked to recruit up to five respondents (i.e., snowball sampling), barring psychology majors, while prioritizing non-college students as potential respondents. Data were collected by an online questionnaire and participants did not receive any incentive for participation. Participants were first presented with a short introduction describing the general aims of the research. Then, they were asked to provide their consent to participate and presented with the questionnaire. A sample of 774 Italian respondents (418 female, *Mage* = 38.4, *SDage* = 13.9) was obtained. A majority (78.8%) of the sample were non-student adults. Among them, 68.5% were employed, 5.1% were retired or houseworkers, and 3.2% were unemployed. The remaining 22.2% of the sample were college students. As for education, 4.4% had a lower secondary school diploma, 45.2% a high school diploma, 39.5% a degree, and 9.4% had a post-graduate qualification. It is important to note that data collection was carried out in the immediate aftermath (April 2018) of the Italian general elections of March 4, 2018. Italian populist parties emerged as politically victorious at the ballots, and a government based on the alliance of two populist parties (i.e., M5S and League) was formed in June 2018. Results of the elections saw the 5 Star Movement as the single most voted list collecting over 32% of votes. It was followed by the League which collected about 18% of the votes, which summed with votes of other parties of the right-wing coalition arrived at about 37%. The Democratic Party (19%) together with other parties of the presented left-wing coalition totalized 23% of preferences. A large part of the electorate did not go to the polls (27%). In the present sample, 23.3% of participants stated that they had voted for the Five Star Movement, 24.4% for the Populist Right-Wing Coalition, 19.9% for the Democratic Party, 12.3% for other left-wing parties, and 12.8% abstained. Although the sampling procedure adopted in the present study did not allow for reaching a representative sample of the Italian population, the voting behavior percentages were not so distant from the results seen in the elections.

### Measures

Unless specified otherwise, response scales were made on a 7-point disagree/agree format.

#### Populist ideology

*Akkerman et al. [6]*. This one-dimensional measure of populist attitude includes six items (*α* = .80).

*Wirth et al. [8]/Schulz et al. [19]*. This scale comprises three dimensions, indexed each by four items: Anti-elitism Attitudes (*α* = .72), Demand for People’s Sovereignty (*α* = .81), and Belief in a Homogeneous and Virtuous People (*α* = .78). It should be noted that some items of this measure overlap with those of the Akkerman’s measure (see Table 2).

**Table 2:** Nodes’ labels and descriptive statistics of the initial network structure of populist ideology.

*Node*	*Dimension*	*Item*	*Author*	*M*	*SD*	*Skewness*	*Kurtosis*
AnMa1_A_W	Anti-Elitism/Manichaeism	Politicians talk too much and take too little action	Wirth/Schulz Akkerman	5.36	1.58	-.773	-.306
ANT1_S	Anti-Elitism	The government is pretty much run by a few big interests looking out for themselves	Castanho Silva	5.14	1.57	-.568	-.565
ANT2_S_r	Anti-Elitism	Government officials use their power to try to improve people’s lives*	Castanho Silva	4.80	1.45	-.327	-.412
ANT3_S	Anti-Elitism	Quite a few of the people running the government are crooked	Castanho Silva	5.12	1.56	-.580	-.353
ANTI1_A	Anti-Elitism	The political differences between the elite and the people are larger than the differences among the people	Akkerman	4.68	1.60	-.344	-.560
ANTI1_W	Anti-Elitism	MPs in Parliament very quickly lose touch with ordinary people	Wirth/Schulz	5.34	1.51	-.920	.347
ANTI2_A	Anti-Elitism	I would rather be represented by an ordinary citizen than by a professional politician	Akkerman	3.79	1.91	.143	-1.103
ANTI2_W	Anti-Elitism	The differences between ordinary people and the ruling elite are much greater than the differences between ordinary people	Wirth/Schulz	4.99	1.51	-.547	-.330
ANTI3_W	Anti-Elitism	People like me have no influence on what the government does	Wirth/Schulz	4.74	1.73	-.368	-.885
HOM1_W	People Homogeneity	Ordinary people all pull together	Wirth/Schulz	4.07	1.65	-.116	-.801
HOM2_W	People Homogeneity	Ordinary people are of good and honest character	Wirth/Schulz	3.62	1.64	.173	-.770
HOM3_W	People Homogeneity	Ordinary people share the same values and interests	Wirth/Schulz	3.80	1.62	.010	-.811
HOM4_W	People Homogeneity	Although the (Italians) are very different from each other, when it comes down to it, they all think the same	Wirth/Schulz	2.96	1.73	.584	-.699
MAN1_S	Manichaeism	You can tell if a person is good or bad if you know their politics	Castanho Silva	2.48	1.66	.960	-.069
MAN2_A	Manichaeism	What people call “compromise” in politics is really just selling out on one’s principles	Wirth/Schulz	4.43	1.73	-.204	-.849
MAN2_S_r	Manichaeism	The people I disagree with politically are not evil*	Castanho Silva	3.04	1.82	.676	-.459
MAN3_S	Manichaeism	The people I disagree with politically are just misinformed	Castanho Silva	3.68	1.80	.079	-.936
SOV1_A_W	People Sovereignty	The politicians in Parliament need to follow the will of the people	Wirth/Schulz Wirth/Schulz	5.45	1.61	-.963	.116
SOV1_S	People Sovereignty	Politicians should always listen closely to the problems of the people	Castanho Silva	5.87	1.42	-1.406	1.569
SOV1_W	People Sovereignty	The people should have the final say on the most important political issues by voting on them directly in referendums	Wirth/Schulz	4.80	1.87	-.506	-.875
SOV2_A_W	People Sovereignty	The people, not the politicians, should make our most important policy decisions	Akkerman Wirth/Schulz	4.31	1.87	-.148	-1.057
SOV2_S_r	People Sovereignty	Politicians don’t have to spend time among ordinary people to do a good job*	Castanho Silva	4.65	1.80	-.352	-1.029
SOV2_W	People Sovereignty	The people should be asked whenever important decisions are taken	Wirth/Schulz	4.50	1.88	-.315	-1.029
SOV3_S	People Sovereignty	The will of the people should be the highest principle in this country’s politics	Castanho Silva	4.98	1.58	-.507	-.502

*Castanho Silva et al.* [7]. The scale includes nine items indexing three distinct dimensions: People-centrism (*α* = .47), Anti-elitism (*α* = .67), and Manichean Outlook (*α* = .31).

#### Political orientation

Political orientation was measured by two items (“In the current Italian political context, how would you describe your political orientation?”; “From a political point of view, you personally feel as a person of…”) reflecting the classical “left–right” dimension of political orientation. Participants answered the two items on a 7-point Likert scale ranging from “Extremely left” to “Extremely right.” Answerers were averaged (*M* = 3.68; *SD* = 1.51, *α* = .89).

#### Ideological attitudes

Participants answered 10 items from the right-wing authoritarianism scale [13] and eight items from the social dominance orientation scale [14]. RWA investigates the inclination to submit to the authorities, to follow conventions and standards of society, and to be hostile toward individuals who do not stick to them (e.g., “The only way our country can get through the crisis ahead is to get back to our traditional values, put some tough leader in power, and silence the troublemakers spreading bad ideas”). SDO assesses the support for the social hierarchy and inequality (e.g., “An ideal society requires some groups to be on top and others to be on the bottom”). Responses to each item were averaged to obtain an overall score whose high values corresponded to high SDO (*M* = 2.47, *SD* = 1.1, *α* = .87) and RWA (*M* = 3.54, *SD* = 1.2, *α* = .85).

#### Self-reported vote

Participants were asked to self-report the party they voted for at the Italian general elections of March 4, 2018. Overall, 23.3% stated that they had voted for the Five Star Movement, 24.4% for the Populist Right-Wing Coalition, 19.9% for the Democratic Party, 12.3% for other left-wing parties, and 12.8% abstained. Self-reported voting was recoded into two dummy variables, the first opposing voters for the M5S (coded 1) to other voters (coded 0); and the second opposing the Populist Right (PRW; coded 1) to other voters (coded 0). Such coding enabled the consideration of the dummies as indicating belongingness to specific electorate clusters and, thus, the comparison of populist and non-populist voters.

#### Anti-immigration attitudes

A single-item measure on the government policies in relation to immigration with four response options was used, ranging from permissive (“Allow irregular immigrants to stay in Italy and apply for citizenship, without penalties”) to restrictive political actions (“Make irregular immigration a crime and expel legal immigrants to their countries of origin”). This item has been borrowed from the American National Election Study (2016) survey. The responses averaged 2.45 (*SD* = 0.79).

### Analytic strategy

#### Network analysis

Network analysis suits the purpose of modeling populist attitudes, ideological attitudes, populist voting, and anti-immigration stances as an integrated system of interconnected beliefs. In respect of classical methodologies, it allows to portray the potential structure of the interplay of psychological variables with the aim of topologically and statistically highlighting its core and peripheral components [38]. These specificities of network analysis are particularly relevant and robust when there are purposes of an exploratory nature both in terms of the factorial structure of a measure [e.g., 39, 40] and of weighted associations of variables within an integrative model [38, 41]. Within a network, observed variables are framed as nodes, and their interplay is quantified through edges representing statistical relationships. In this study, the edges between nodes (e.g., items of populist attitude measures) were expressed in terms of partial correlations. Partial correlations are particularly useful in the context of network analysis, as problems of spurious correlation due to intercorrelation among variables are efficiently dealt with [42]. An edge between two nodes expressed as a partial correlation indicates conditional dependence between the nodes. Conversely, when two nodes are not linked, they are conditionally independent given all other nodes in the network and thus cannot interact directly. For this reason, partial correlations can be indicative of potential causal pathways. Indeed, partial correlation networks are thought of as highly exploratory hypothesis-generating structures, indicative of potential causal effects [43].

Network models involving partial correlations [Gaussian Graphical Model, GGM; 44] follow the general principles of specificity and sensitivity. Increasing the model’s specificity reduces the edges between nodes, resulting in a sparser network model. On the other hand, increasing the sensitivity makes the model less parsimonious, rising the risk of encountering unreliable edges (i.e., a denser network model). To find a balance between the specificity and the sensitivity of a model, a regularization is implemented through the graphical Least Absolute Shrinkage and Selection Operator [*glasso*; 45] and the Extended Bayesian Information Criterion [EBIC; 46]. The *glasso* forced the edges’ estimates of minor importance to 0 and excluded them from the model. The EBIC is used for selecting the best-fitting regularized partial correlations network [47]. The *EBICglasso method*, combining the *glasso* algorithm with the EBIC, proceeds iteratively to estimate models with different combinations among nodes and concludes by selecting the most informative one [41]. It returns a pairwise Markov random field [PMRF; 42] in which nodes are connected by undirected edges (i.e., all variables are free to exert mutual influence) indicating their conditional dependence. In other words, the analysis yields a network of *regularized* partial correlations between nodes.

In the present research, the estimation of the first network model aimed to examine the structure of populist ideology by adopting an *Exploratory Graph Analysis* approach [39]. It involves the implementation of the above-mentioned *EBICglasso method* for the estimation of the network structure. The number of dimensions was established by the *walktrap* algorithm [48]. The algorithm implements “random walks” or a stochastic number of steps from one node, across one edge, to another. The latent dimensions (or communities) are determined within four steps of the random walks. Thus, nodes are grouped into a community if they are connected to each other within four passes across their edges [40]. The predetermined number of four steps derives from previous simulation studies that demonstrated that the *walktrap* algorithm outperforms other community detection algorithms for weighted networks using four steps [40, 49, 50]. The analysis was carried out using the *EGAnet* R package [51], which enabled the implementation of parametric bootstrap (1000 iterations) to assess the stability of the network’s dimensions and the robustness of each item’s placement within those dimensions [52].

The other two network models aimed at investigating the centrality of the distinct populist ideology dimensions (retrieved from the EGA), as well as their interplay with ideological attitudes, anti-immigration attitudes, and populist voting. Even for these models, the network structures were estimated with the *EBICglasso method*. The relevance of the nodes within their network was investigated through three *centrality* indices [42, 53, 54]. *Closeness* corresponds to the inverse of the sum of the focal node distance from all other nodes in the network, in other words, the indirect proximity of a node to the others. *Betweenness* is defined as the number of geodesics among any two nodes passing through the focal one; that is, the number of edges that connect two nodes passing through the focal node. *Strength* is defined as the sum of the edge-weights in absolute value—namely, the magnitude of the focal node’s connections with the other nodes.

The accuracy of the network models and of the resulting centrality indices was tested by following the step-by-step indications provided by Epskamp et al. [43]. First, non-parametric bootstrap (5,000 resampling) was run to draw bootstrapped CIs and assess edge-weights’ accuracy. Then, bootstrapped difference tests between edge-weights or centrality indices were performed to investigate whether they differed significantly from each other. Finally, the stability of centrality indices was evaluated through the *correlation stability coefficient* (*CS-coefficient*) across different portions of the sample. *CS-coefficient* was computed by means of case-dropping bootstrap (5,000 resampling). The RStudio graphical interface [55] was employed for analyses using the *qgraph* [38] and *bootnet* package [43].

## Results

### The network of populist ideology

#### Exploratory graph analysis

The structure of populist ideology was examined through an *exploratory graph analysis* (EGA). Before proceeding with the EGA, negatively worded items were reversed and the normality of distribution for the entire pool of items was tested by checking whether skewness and kurtosis parameters were within the limits of ±2.00 [56]. As shown in Table 2, all the items did not present normality issues and therefore were included in the EGA.

**Fig 1:**
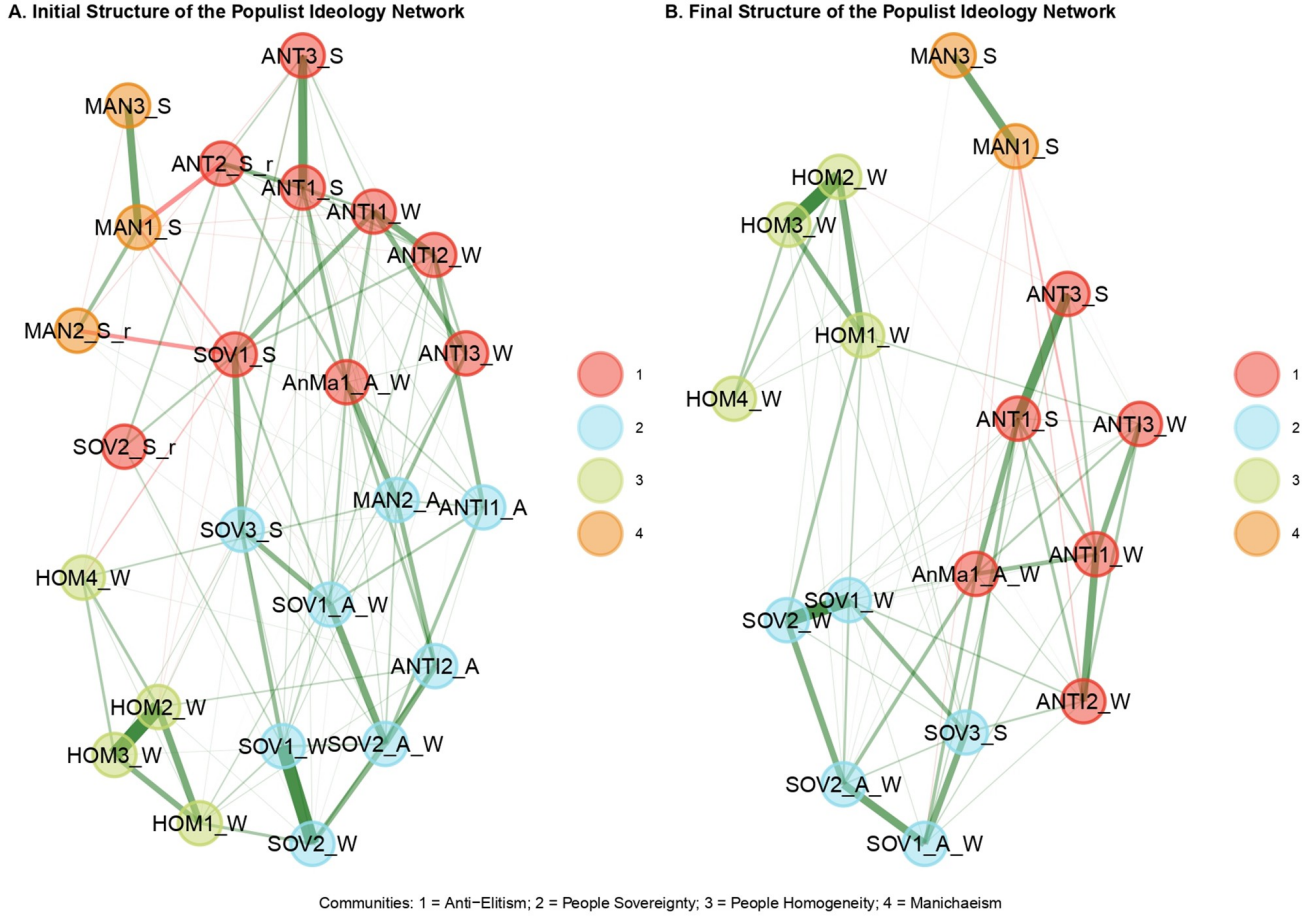
Network structure of populist ideology. (A) Initial solution of populist ideology network resulting from the EGA model. (B) Final solution of populist ideology network after removing unstable items from the EGA model.

**Fig 2:**
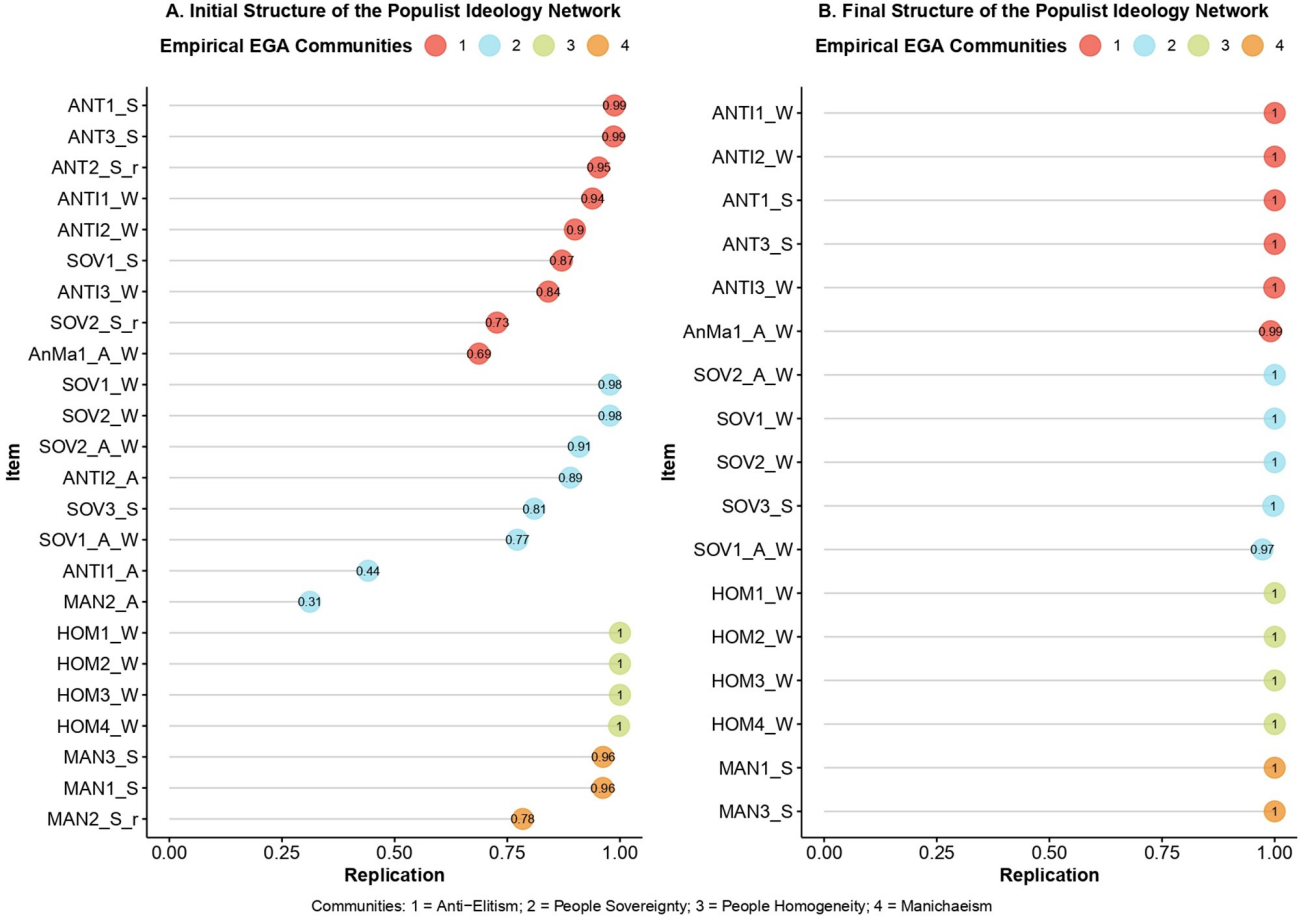
Items’ stability across the initial and final structure of populist ideology network. (A) Items’ stability results from the EGA model of the initial solution of populist ideology network structure. (B) Items’ stability results from the EGA model of the final solution of populist ideology network.

Fig 1A shows the resulting network structure of the populist ideology including the entire pool of populist attitude items. The network consists of 129 non-zero edges out of 276 possible edges. The constitutive dimensions of the populist ideology (i.e., People Sovereignty = azure nodes; Anti-elitism = red nodes; People Homogeneity = green nodes; Manichaeism = orange nodes) suggested in the literature emerge clearly. Most of the items designated for each dimension were conditionally dependent on each other, forming circumscribed clusters (or communities) within the network. To assess the stability of the emerged dimensions, descriptive statistics of the network structure’s dimensions across bootstrap replicate samples were explored. They revealed that the median of the dimensions was 4, reflecting the empirical EGA model, but with a wide confidence interval (*n*.*Boots* = 1000, *median*.*dim* = 4, *SE*.*dim* = 0.57, *95%CI* = 2.88, 5.12). This slight lack of accuracy of estimated dimensions was also corroborated by the frequency of different dimensional solutions across replicated samples: the 4-dimensional solution was found 73% of the time (i.e., 730 of 1000 bootstrap replicates) while a 5-dimensional solution was found 20% of the time. To get a better understanding of which communities were unstable, their specific stability was computed. People Sovereignty and Anti-Elitism resulted to be the most unstable dimensions (*f* = 0.49; *f* = 0.27, respectively), Manichaeism showed only slight instability (*f* = 0.77), while People Homogeneity proved to be stable (*f* = 0.99). The instability of these dimensions was confirmed when looking at the item stability values in the empirical dimensions (Fig 2A). It emerged clearly that some of the items from People Sovereignty, Manichaeism, and Anti-Elitism were unstable or did not load on their theoretically assigned dimension. Specifically, the items “ANTI1_A” and “MAN2_A” loaded on People Sovereignty, while they were not theoretically designed for it. Both items showed poor stability values, indicating their cross loadings between different dimensions across bootstrap samples. The “MAN2_S_r” item showed a slightly poor stability value, though associated with its theoretical dimension of Manichaeism. It also showed a negative, albeit weak, relationship with another item belonging to the same dimension, thus resulting as the problematic one. The items “SOV1_S” and “SOV2_S_r” loaded on Anti-Elitism while they were theoretically destinated to the People Sovereignty dimension. Finally, the item “ANT2_S_R”, although resulting in its dimension, showed strongly negative associations with other items included in the analysis (in particular with the “MAN1_S” item), thus proving to be problematic. These unstable items were clearly causing problems with the consistency of the populist attitude dimensions. Therefore, the analysis was re-performed after removing all the problematic items.

The final structure of populist ideology is presented in Fig 1B. The network consisted of 75 non-zero edges out of 136 possible edges. Even in this final solution, the constitutive dimensions of populist ideology raised clearly. EGA results showed the median value of 4 with a standard error of 0. Thus, the 4 dimensions emerged 100% of the time across the 1000 bootstrap samples. As shown in Fig 2B all the retained items suited excellently in their theoretical dimensions. Thus, the final dimension of People Sovereignty consisted of the nodes “SOV1_A_W”, “SOV2_A_W”, “SOV1_W”, “SOV2_W”, “SOV3_S” (*M* = 4.81, *SD* = 1.36, *α* = .83). Anti-elitism was composed by the nodes “AnMa1_A_W”, “ANT1_S”, “ANT1_W”, “ANT2_W”, “ANT3_S”, “ANT3_W” (*M* = 5.11, *SD* = 1.10, *α* = .79). People Homogeneity consisted of all the four nodes named “HOM” in Table 2 (*M* = 3.61, *SD* = 1.28, *α* = .78). The Manichean outlook dimension was composed of the “MAN1_S” and “MAN3_S” (*M* = 3.01, *SD* = 1.41, *α* = .50). A detailed description of the nodes is shown in Table 2, while partial correlations matrices of both EGA models can be found in the supplementary material (Matrix 1 and Matrix 2 in S1 File).

#### Populist narratives’ centrality

Aiming to describe the centrality of the distinct populist dimensions within the populist ideology network, an aggregated scale-level model resulting from the final EGA has opted. Due to an imbalance in the number of items belonging to each dimension and therefore more connected to each other, an item-level model risked inflating the estimate of centrality indices. The aggregated scale-level model thus enabled a more accurate estimation of the interested indices.

**Fig 3:**
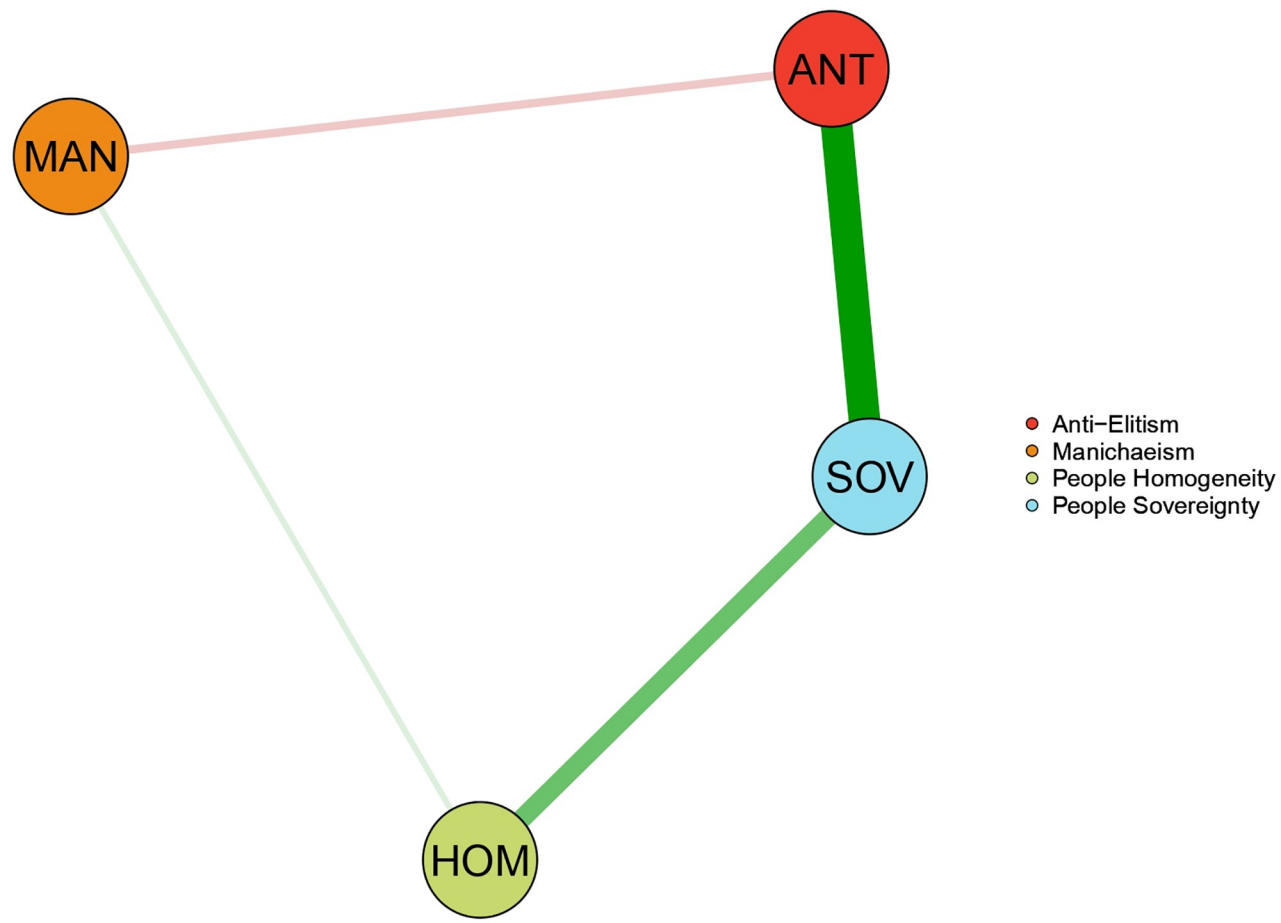
Network of populist ideology dimensions. It reports the network model investigating populist narratives’ centrality.

**Fig 4:**
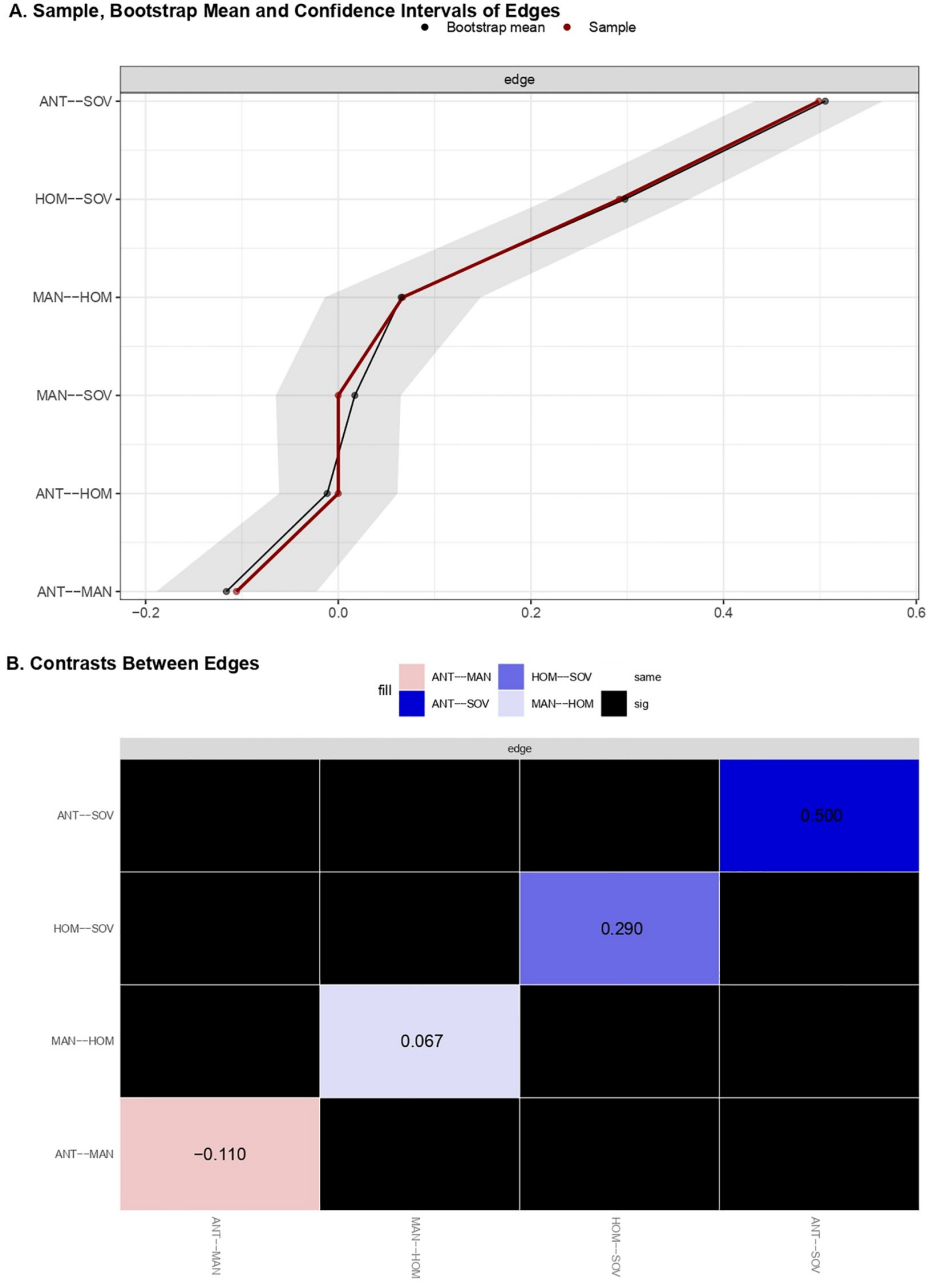
Edge weights of the network of populist ideology dimensions. (A) Sample, bootstrap means and confidence intervals of edge weights resulting from the network model investigating populist narratives’ centrality. (B) Bootstrap differences of edges weights resulting from the network model investigating populist narratives’ centrality.

Fig 3 shows the estimated network of populist ideology dimensions consisting of 4 out of 6 possible edges. Table 3 summarized the associations that emerged in the network structure. People Sovereignty (i.e., SOV) appeared strongly and positively related to Anti-elitism (i.e., ANT) and People Homogeneity (i.e., HOM) and was not associated with Manichaeism (i.e., MAN). The MAN dimension indeed appeared as peripheral, showing only an unstable positive edge with HOM and a negative relation with ANT (Fig 4A). The ANT and HOM dimensions emerged as not related to each other and thus they interacted indirectly through their respective strong associations with SOV. Indeed, the association between SOV and ANT was the strongest of the network. It was followed by the association between SOV and HOM which emerged as significantly stronger than the remaining associations (Fig 4B).

**Table 3:** Sample and bootstrap mean of edge-weights of the populist ideology dimensions network.

	*Estimates (r*_*partial*_ *)*			*95%CI*	
*Edge*	*Sample*	*Mean*	*SD*	*Lower*	*Upper*
ANT--HOM	0.000	-0.012	0.031	-0.061	0.061
ANT--MAN	-0.106	-0.116	0.042	-0.189	-0.022
ANT--SOV	0.499	0.505	0.033	0.432	0.565
HOM--SOV	0.292	0.298	0.036	0.220	0.364
MAN--HOM	0.067	0.065	0.040	-0.014	0.148
MAN--SOV	0.000	0.017	0.032	-0.065	0.065

**Fig 5:**
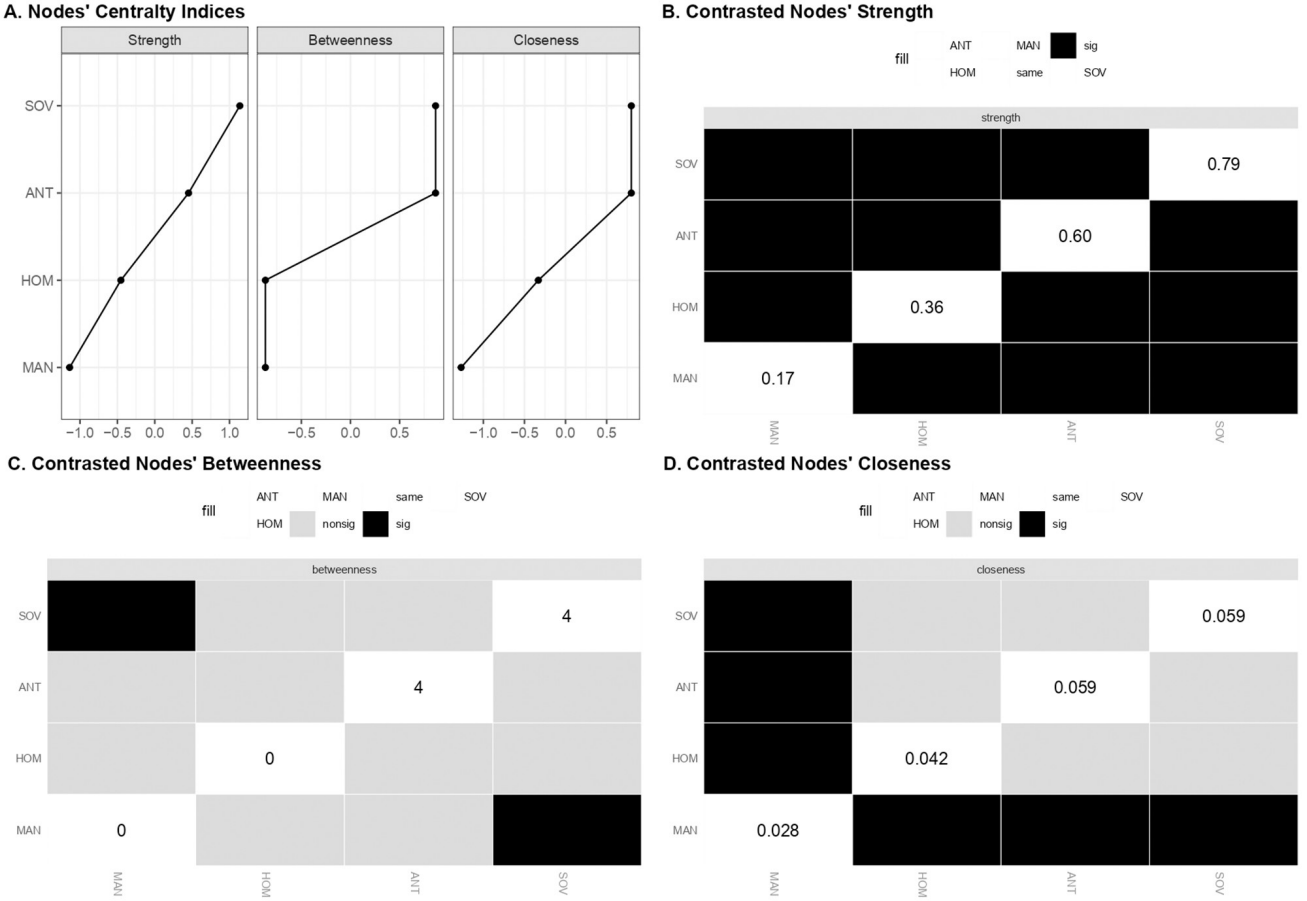
Estimates and bootstrap differences of centrality indices of the populist ideology dimensions network. (A) Standardized values of centrality indices for each populist ideology dimension. (B) Bootstrap differences between nodes for the strength centrality index. (C) Bootstrap differences between nodes for the betweenness centrality index. (D) Bootstrap differences between nodes for the closeness centrality index. Fig 5B-5D report sample estimates on the diagonal, while bootstrap differences in the black and gray boxes. Black boxes indicate significant differences based on bootstrap mean estimates.

The whole edges’ pattern provided a first indication of the central role of People Sovereignty in the ideological space of populism. Such a role was corroborated by the estimates of centrality indices (Table 4). As shown in Fig 5A, the SOV dimension displayed the highest values of strength and, together with ANT, the highest value of betweenness and closeness. HOM and MAN had lower values on all centrality indices, with MAN showing values close to 0. Further support was obtained by examining differences in bootstrap means of centrality indices for apparent central and peripheral nodes. The nodes’ strength estimates differed significantly from each other, with SOV showing the highest value. SOV was followed by ANT, which in turn differed significantly from HOM and MAN. Even the strength of HOM was significantly greater than that of MAN (Fig 5B). In terms of betweenness, the analysis revealed less evident differences between the estimated scores. Although SOV and ANT showed the same sample value, the bootstrap means and the related contrasts revealed that the SOV was the node with the highest value, but also that it differed significantly only from MAN and not from ANT and HOM (Fig 5C). A similar result was obtained when the bootstrap means of closeness were contrasted. SOV and ANT resulted to be the most relevant nodes. However, the unique node showing a significantly lower value than the others was MAN (Fig 5D). The analysis thus indicated People Sovereignty as the most relevant dimension in terms of strength. It was the dimension with the strongest relationships within the network. As regards closeness and betweenness, People Sovereignty shared its relevance with People Homogeneity and, above all, with Anti-Elitism. These three dimensions were similar in terms of indirect proximity with the other nodes of the network and in playing a bridging role between them. The most disconnected node was the dimension of Manichaeism showing the worst values in all centrality indices. Overall, results suggested the dimension of People Sovereignty as being more central within the structure of populist ideology, but also that it shared its relevance with Anti-elitism and People Homogeneity. Manichaeism was instead peripheral in the populist narrative space.

**Table 4:** Sample and bootstrap mean of centrality indices of the populist ideology dimensions network.

	Strength					Betweenness					Closeness				
	*Estimates*			*95%CI*		*Estimates*			*95%CI*		*Estimates*			*95%CI*	
Node	*Sample*	*Mean*	*SD*	*Low*.	*Up*.	*Sample*	*Mean*	*SD*	*Low*.	*Up*.	*Sample*	*Mean*	*SD*	*Low*.	*Up*.
ANT	0.604	0.635	0.075	0.454	0.755	4	2.860	1.335	1.330	6.670	0.059	0.061	0.014	0.031	0.087
HOM	0.359	0.377	0.077	0.205	0.512	0	0.226	0.787	-1.573	1.573	0.042	0.047	0.010	0.022	0.062
MAN	0.173	0.200	0.084	0.005	0.340	0	0.000	0.000	0.000	0.000	0.028	0.031	0.009	0.010	0.046
SOV	0.790	0.821	0.069	0.652	0.929	4	3.126	0.993	2.014	5.986	0.059	0.061	0.014	0.032	0.086

**Fig 6:**
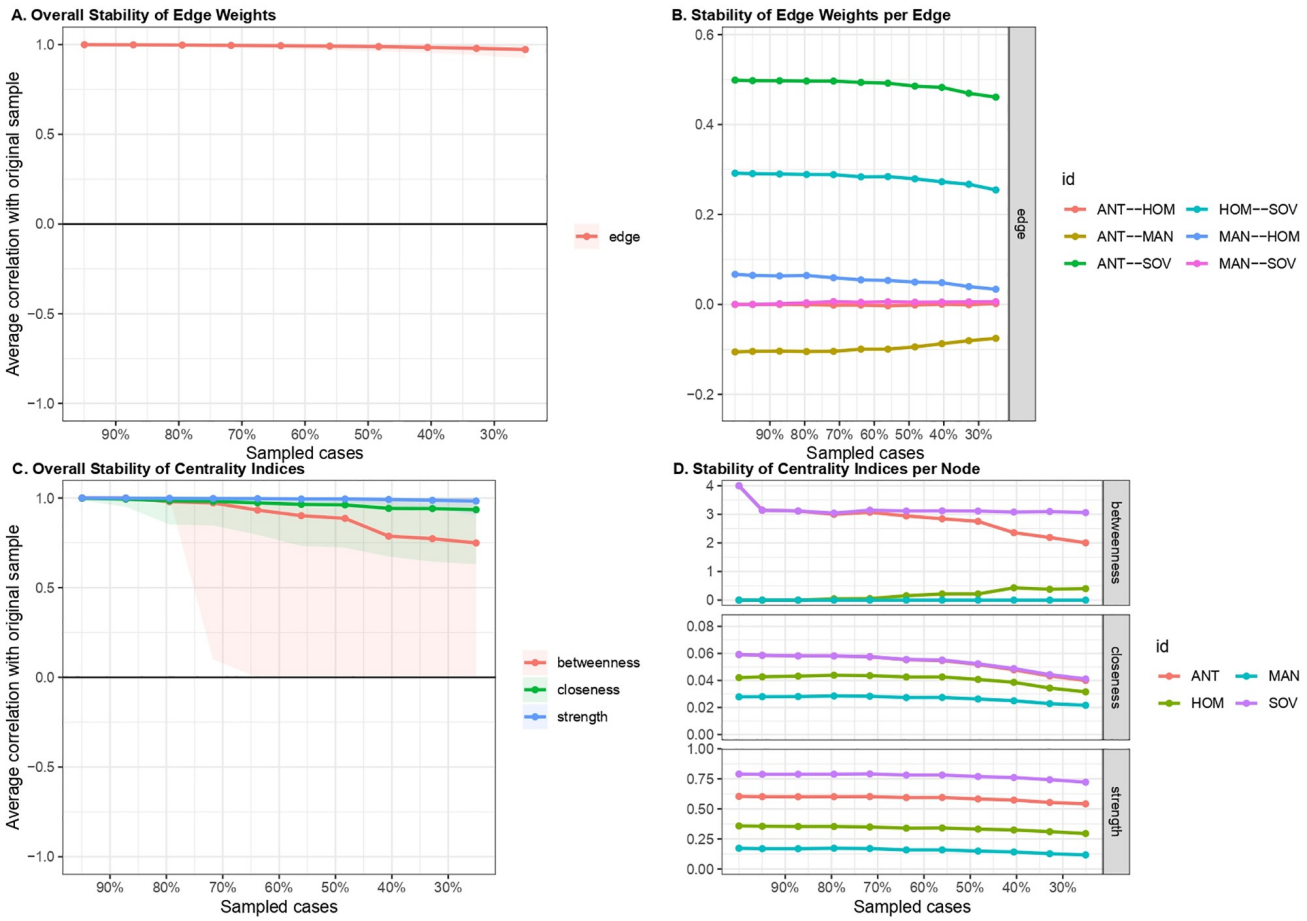
Stability of edge weights and centrality indices of populist ideology dimensions network. (A) Overall stability of edge weights. (B) Stability of each edge weight. (C) Overall stability of estimated centrality indices. (D) Stability of estimated centrality indices for each node.

Finally, the stability of edges and centrality indices was investigated by estimating the populist ideology network structure based on subsets of the data. Estimated edges (Fig 6A and 6B) and centrality indices (Fig 6C and 6D) kept a certain degree of stability even when a large portion of the sample was dropped out. Specifically, edges and strength parameters remained stable even when most of the sample was dropped; instead, estimates for betweenness and closeness showed a slight slope when 20% of the sample was dropped. Stability was quantified using the *CS-coefficient*, which indicated that estimated edges (*CS*(*cor* = 0.7) = 0.75) and strength (*CS*(*cor* = 0.7) = 0.75) were highly stable under subsetting cases. The *CS-coefficient* of betweenness and closeness were equal to 0.29 and 0.38, respectively. This may be due to the presence of few nodes and edges in the network [41]. However, as indicated by Epskamp et al. [43], to interpret centrality differences, the CS-coefficient should not be below 0.25, and preferably above 0.5.

#### RWA and SDO within the populist ideology network

**Fig 7:**
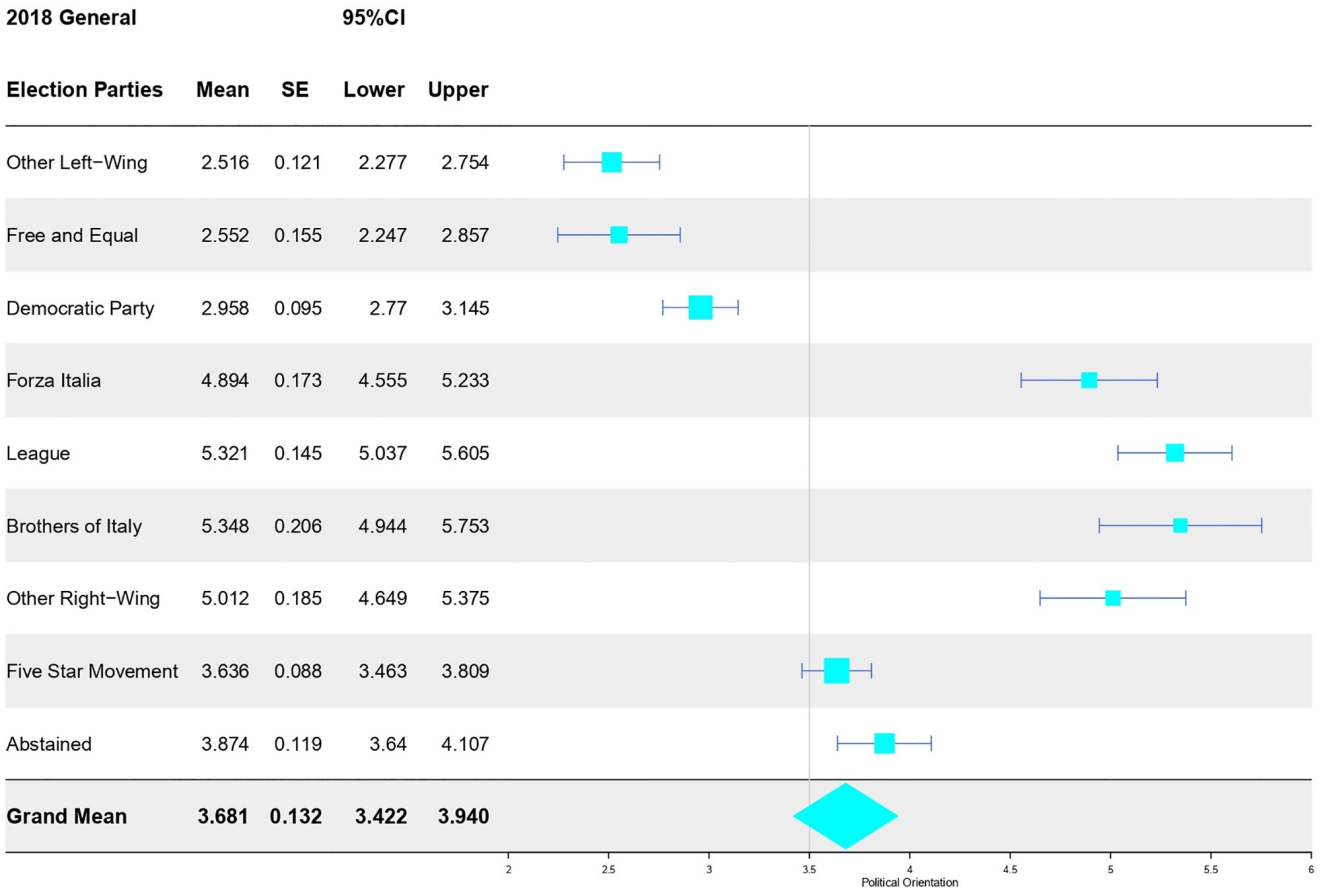
Forest plot of political orientation’s average scores across voters of candidate parties in the 2018 general election.

*Positioning of populist parties on the left-right continuum of political orientation*. As a preliminary step, the ranking of the different political groups in terms of left-to-right orientation was explored. This aimed to check whether the right-wing populist voters scored differently from those of M5S, which should represent the “*valence*” variety of populism. A between-subjects ANOVA confirmed the existence of omnibus unsurprising differences in self-reported political orientation (*F*_[8, 773]_ = 62.53, *p* < .001, *η*^*2*^ = .40). As shown in Fig 7, averages closely conformed to Zulianello’s (2020) classification. *Bonferroni post-hoc comparisons* indicated that Forza Italia’s voters (i.e., right-wing populist voters with a lower score of right-wing political orientation) showed a mean score that was significantly shifted to the right side of the continuum, compared to the populist counterpart of M5S voters (*M*_*diff*_ = 1.26, *se* = .19, *p* < .001, *95%CI* = 0.635, 1.880). M5S voters showed intermediate value, placed exactly in the middle of the left–right spectrum, and—importantly—that was significantly to the right of Democratic Party voters (i.e., a classic social democrat left-wing party: *M*_*diff*_ = 0.68, *se* = .13, *p* < .001, *95%CI* = 0.261, 1.095). The average for M5S voters overlapped with abstainers’ average score (*M*_*diff*_ = -0.24, *se* = .15, *p* = .99, *95%CI* = -0.713, 0.238). These analyses photograph the placement of populist parties on the classic left-right continuum, corroborating the notion that the M5S reflects fairly well the notion of valence populism, being distinct from right-wing populism, as well as different from classical left-wing equality-oriented parties.

### Bivariate correlations

As a further descriptive step, correlations among investigated variables were computed (Table 5). Significant associations of ideological attitudes with populist ideology dimensions, voting, and anti-immigration attitudes were found. RWA was positively associated with Anti-elitism and People sovereignty, which appeared to be core narratives of populist ideology. Instead, SDO showed a negative zero-order association with both these dimensions. RWA showed no association with Manichaeism and a positive association with People Homogeneity, while SDO showed to be positively related to Manichaeism and unrelated to People Homogeneity. Ideological attitudes correlated positively with anti-immigration attitudes, political orientation, and PRW voting. M5S voting, instead, showed non-significant associations with RWA and SDO.

**Table 5:** Intercorrelations among examined variables.

Variables	1	2	3	4	5	6	7	8	9
1. Right-Wing Authoritarianism	-								
*[95%CI]*									
2. Social Dominance Orientation	.23***	-							
*[95%CI]*	[.16, .30]								
3. People Sovereignty	.30***	-.25***	-						
*[95%CI]*	[.23, .36]	[-.32, -.19]							
4. Anti-Elitism	.25***	-.23***	.55***	-					
*[95%CI]*	[.19, .32]	[-.30, -.17]	[.50, .60]						
5. Manichaeism	.05	.16***	-.01	-.14***	-				
*[95%CI]*	[-.03, .12]	[.09, .23]	[-.09, .06]	[-.21, -.07]					
6. People Homogeneity	.38***	.01	.36***	.15***	.08*	-			
*[95%CI]*	[.31, .43]	[-.06, .08]	[.30, .42]	[.08, .22]	[.01, .15]				
7. Five Star Movement Vote	.06	-.05	.21***	.18***	-.04	.09*	-		
*[95%CI]*	[-.01, .13]	[-.12, .02]	[.14, .28]	[.11, 25]	[-.11, .03]	[.02, .16]			
8. Populist Right-Wing Vote	.35***	.21***	.05	.07	-.06	.11**	-.31***	-	
*[95%CI]*	[.28, .41]	[.14, .27]	[-.02, .12]	[-.01, .14]	[-.13, .09]	[.04, .18]	[-.37, -.25]		
9. Political Orientation	.54***	.31***	.11**	.11**	-.08*	.21***	-.02	.55***	-
*[95%CI]*	[.49, .59]	[.24, .37]	[.04, .18]	[.04, .18]	[-.15, -.01]	[.14, .27]	[-.09, .05]	[.50, .60]	
10. Anti-Immigration Attitudes	.32***	.19***	.14***	.11**	-.002	.11**	.09*	.17***	.35***
*[95%CI]*	[.26, .38]	[.12, .26]	[.07, .20]	[.04, .18]	[-.07, .07]	[.04, .18]	[.02, .16]	[.10, .23]	[.28, .41]

Note:

* p < .05,

** p < .01,

*** p < .001.

#### The integrated beliefs system network

**Fig 8:**
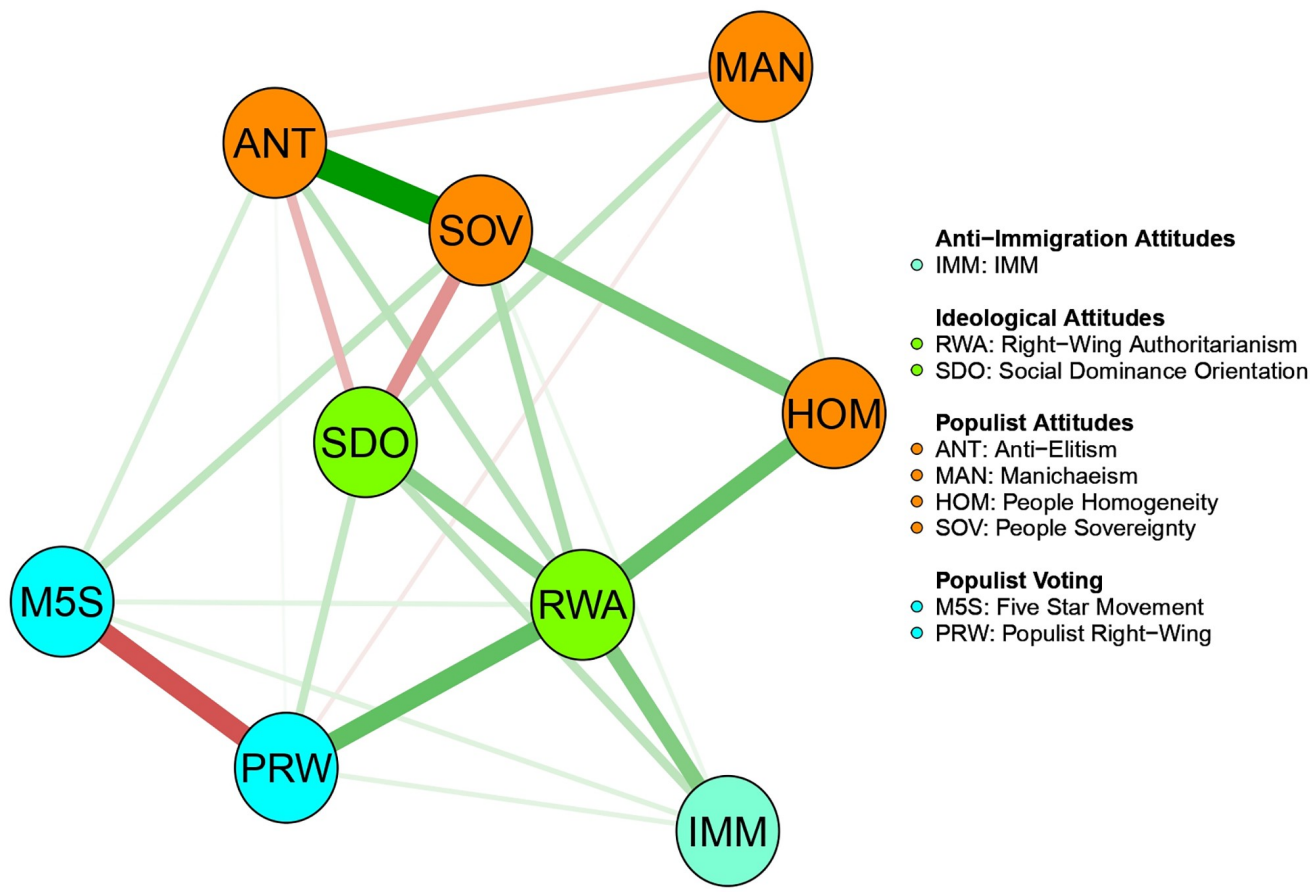
The Integrated beliefs system network. It reports the network model including Ideological attitudes, Anti-immigration attitudes, Populist Ideology and Voting.

Fig 8 shows the resulting network structure of the beliefs system including ideological and populist attitudes, self-reported voting, and anti-immigration attitudes. The network model consisted of 25 non-zero edges out of 36 possible edges.

**Fig 9:**
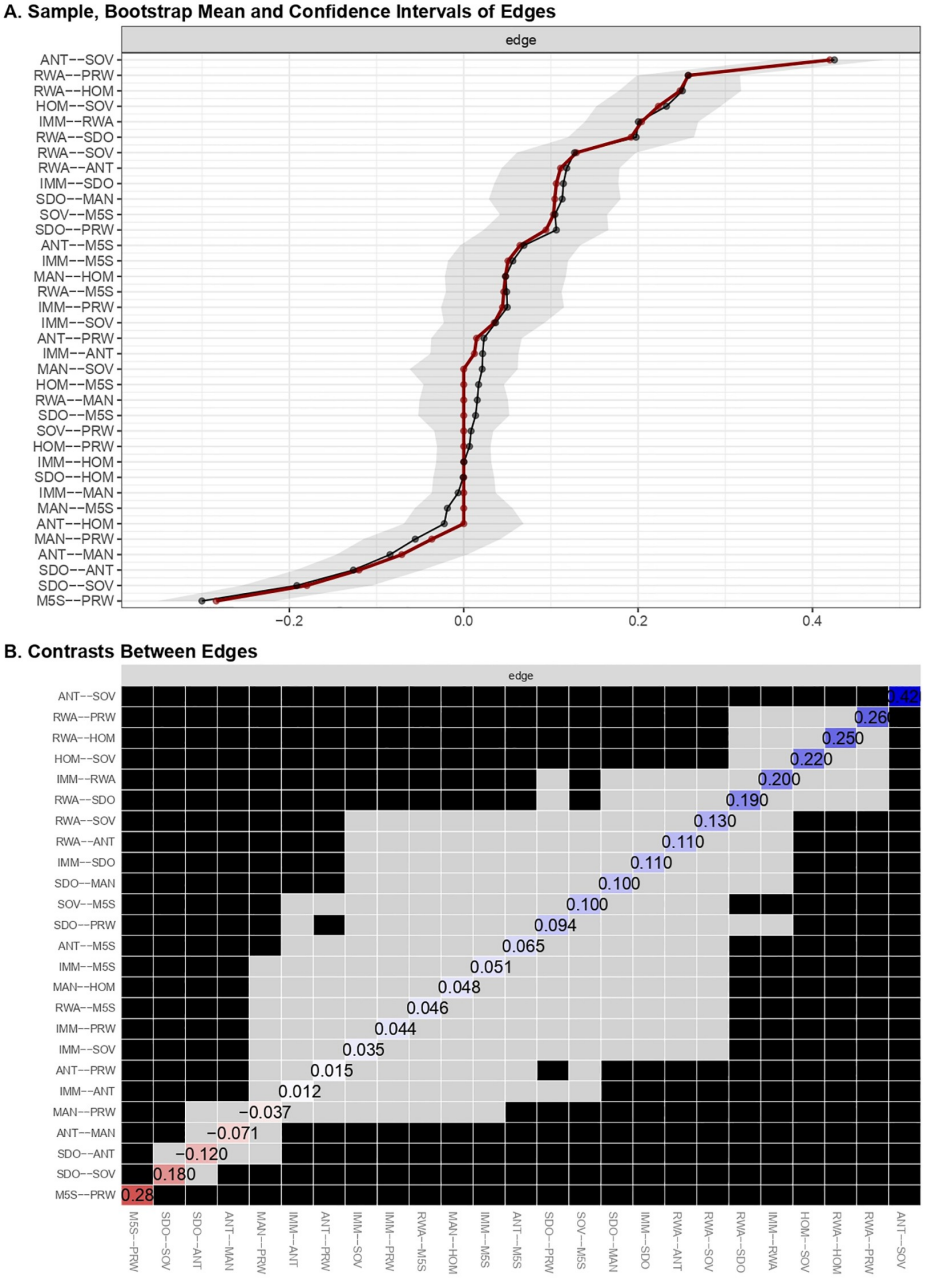
Edge weights of the integrated beliefs system network. (A) Sample, bootstrap means and confidence intervals of edge weights resulting from the integrated beliefs system network. (B) Bootstrap differences of edges weights resulting from the integrated beliefs system network.

Focusing on the dimensions of populist ideology, People Sovereignty appeared to play a pivotal role in connecting other populist dimensions even in this wider network. SOV maintained robust associations with Anti-elitism and People Homogeneity (Fig 9A). The edge between SOV and ANT was the significantly strongest of the network (see Fig 9B). Thus, People Sovereignty held together the other two dimensions of populist ideology. The Manichean outlook appeared again peripheral. It was substantially unrelated to any dimensions of populism and to other variables included in the network, apart from an association with SDO (Table 6). Consistent with the correlation summarized above, SDO was also negatively associated (red edge in Fig 8) with People Sovereignty and Anti-elitism. The stronger the endorsement of a hierarchical division of society, the weaker the tendency to exalt the “General Will” of the People and to delegitimize leading social groups. Instead, RWA showed reliable direct connections with the populist dimensions of People Sovereignty, Anti-elitism, and People Homogeneity (Table 6). It may be concluded that motives related to conservatism and tradition appeared relevant to populist ideology tenets.

**Table 6:** Sample and bootstrap mean of edge-weights of the integrated beliefs system network including ideological attitudes, anti-immigration attitudes, populist ideology and voting.

	*Estimates (r*_*partial*_ *)*			*95%CI*	
*Edge*	*Sample*	*Mean*	*SD*	*Lower*	*Upper*
ANT--HOM	0.000	-0.022	0.034	-0.069	0.069
ANT--M5S	0.065	0.069	0.034	-0.004	0.133
ANT--MAN	-0.071	-0.085	0.037	-0.146	0.004
ANT--PRW	0.015	0.023	0.026	-0.037	0.067
ANT--SOV	0.420	0.425	0.031	0.357	0.482
HOM--M5S	0.000	0.017	0.023	-0.046	0.046
HOM--PRW	0.000	0.007	0.015	-0.031	0.031
HOM--SOV	0.223	0.233	0.036	0.152	0.295
IMM--ANT	0.012	0.022	0.025	-0.038	0.063
IMM--HOM	0.000	0.000	0.016	-0.032	0.032
IMM--M5S	0.051	0.056	0.034	-0.018	0.120
IMM--MAN	0.000	-0.006	0.018	-0.037	0.037
IMM--PRW	0.044	0.050	0.035	-0.026	0.115
IMM--RWA	0.204	0.200	0.033	0.139	0.269
IMM--SDO	0.106	0.114	0.036	0.035	0.177
IMM--SOV	0.035	0.037	0.029	-0.023	0.093
M5S--PRW	-0.284	-0.300	0.034	-0.351	-0.217
MAN--HOM	0.048	0.048	0.035	-0.022	0.117
MAN--M5S	0.000	-0.019	0.028	-0.056	0.056
MAN--PRW	-0.037	-0.056	0.039	-0.115	0.042
MAN--SOV	0.000	0.021	0.031	-0.062	0.062
RWA--ANT	0.111	0.118	0.034	0.043	0.179
RWA--HOM	0.248	0.251	0.035	0.178	0.318
RWA--M5S	0.046	0.049	0.033	-0.020	0.112
RWA--MAN	0.000	0.016	0.025	-0.051	0.051
RWA--PRW	0.258	0.257	0.029	0.199	0.317
RWA--SDO	0.192	0.198	0.036	0.120	0.264
RWA--SOV	0.129	0.127	0.034	0.061	0.198
SDO--ANT	-0.120	-0.127	0.036	-0.193	-0.048
SDO--HOM	0.000	-0.001	0.017	-0.035	0.035
SDO--M5S	0.000	0.014	0.026	-0.052	0.052
SDO--MAN	0.104	0.113	0.038	0.029	0.180
SDO--PRW	0.094	0.106	0.036	0.023	0.166
SDO--SOV	-0.180	-0.191	0.037	-0.254	-0.105
SOV--M5S	0.103	0.105	0.031	0.041	0.165
SOV--PRW	0.000	0.008	0.017	-0.034	0.034

Anti-immigration attitudes were directly linked only with RWA and SDO, whilst it was not directly connected to populist dimensions and voting (Table 6). The edge with RWA was significantly stronger than those with all other nodes, but not stronger vis-à-vis SDO (see Table 7). PRW dummy was only directly related to both ideological attitudes, highlighting the strongest edge with RWA in respect of all other nodes (Table 7), and just weakly and unstably linked to anti-immigration attitudes. PRW voting was not related to any dimension of populist ideology (Table 6). M5S voting resulted only robustly associated with the SOV dimension of populist ideology. It had weak edges with RWA and IMM, which resulted to be unstable across bootstrap mean estimates, and no connection to SDO (Table 6). However, bootstrap difference-tests (Fig 9B) indicated that the strength of such association did not differ statistically from those of the M5S dummy with Anti-elitism, RWA (interestingly), and anti-immigration attitudes (see Table 7).

**Table 7:** Pairwise contrasts of edge-weights’ strength for anti-immigration attitudes and populist voting.

*Focal Node*	*Focal Edge*	*Contrasted Edge*	*95%CI*	
			*Lower*	*Upper*
Anti-Immigration Attitudes	RWA--IMM	SDO--IMM	-.187	.024
	RWA--IMM	SOV--IMM	-.252	-.065
	RWA--IMM	ANT--IMM	-.258	-.088
	RWA--IMM	MAN--IMM	-.286	-.136
	RWA--IMM	HOM--IMM	-.278	-.124
	RWA--IMM	PRW--IMM	-.248	-.039
	RWA--IMM	M5S--IMM	-.238	-.047
Five Star Movement Voting	SOV--M5S	SDO--M5S	-.157	-.014
	SOV--M5S	ANT--M5S	-.139	.071
	SOV--M5S	RWA--M5S	-.140	.040
	SOV--M5S	MAN--M5S	-.220	-.051
	SOV--M5S	HOM--M5S	-.160	-.006
	SOV--M5S	PRW--M5S	-.500	-.311
	SOV--M5S	IMM--M5S	-.134	.042
Populist Right-Wing Voting	RWA--PRW	SDO--PRW	-.250	-.052
	RWA--PRW	SOV--PRW	-.314	-.173
	RWA--PRW	ANT--PRW	-.307	-.152
	RWA--PRW	MAN--PRW	-.415	-.224
	RWA--PRW	HOM--PRW	-.315	-.176
	RWA--PRW	IMM--PRW	-.300	-.101
	RWA--PRW	M5S--PRW	-.655	-.460

*Note*. Contrasts are based on bootstrap mean estimates of the edges.

Overall, the network’s edge weights suggested interesting potential patterns of indirect associations, where the central role played by ideological attitudes in relating the other nodes within the network emerged quite clearly. SDO and RWA were the unique intermediate links between PRW voting and anti-immigration. RWA was also the unique node connecting the dimensions of populist ideology with preference for stricter immigration policies. Through its association with SOV, ANT, and HOM, RWA linked the core of populist ideology to anti-immigration attitudes. Moreover, the association of RWA with People Sovereignty was essential in bringing about the potential indirect pattern of associations regarding M5S voting. M5S was stably linked solely to the dimension of SOV. This association allowed M5S voting to be indirectly associated with RWA and, therefore, to relate to anti-immigration attitudes in an indirect, serial mediated, fashion. Thus, RWA appeared to play a promising bridging role in channeling the association between M5S voting and People Sovereignty toward the assumption of hostile attitudes about immigration.

**Fig 10:**
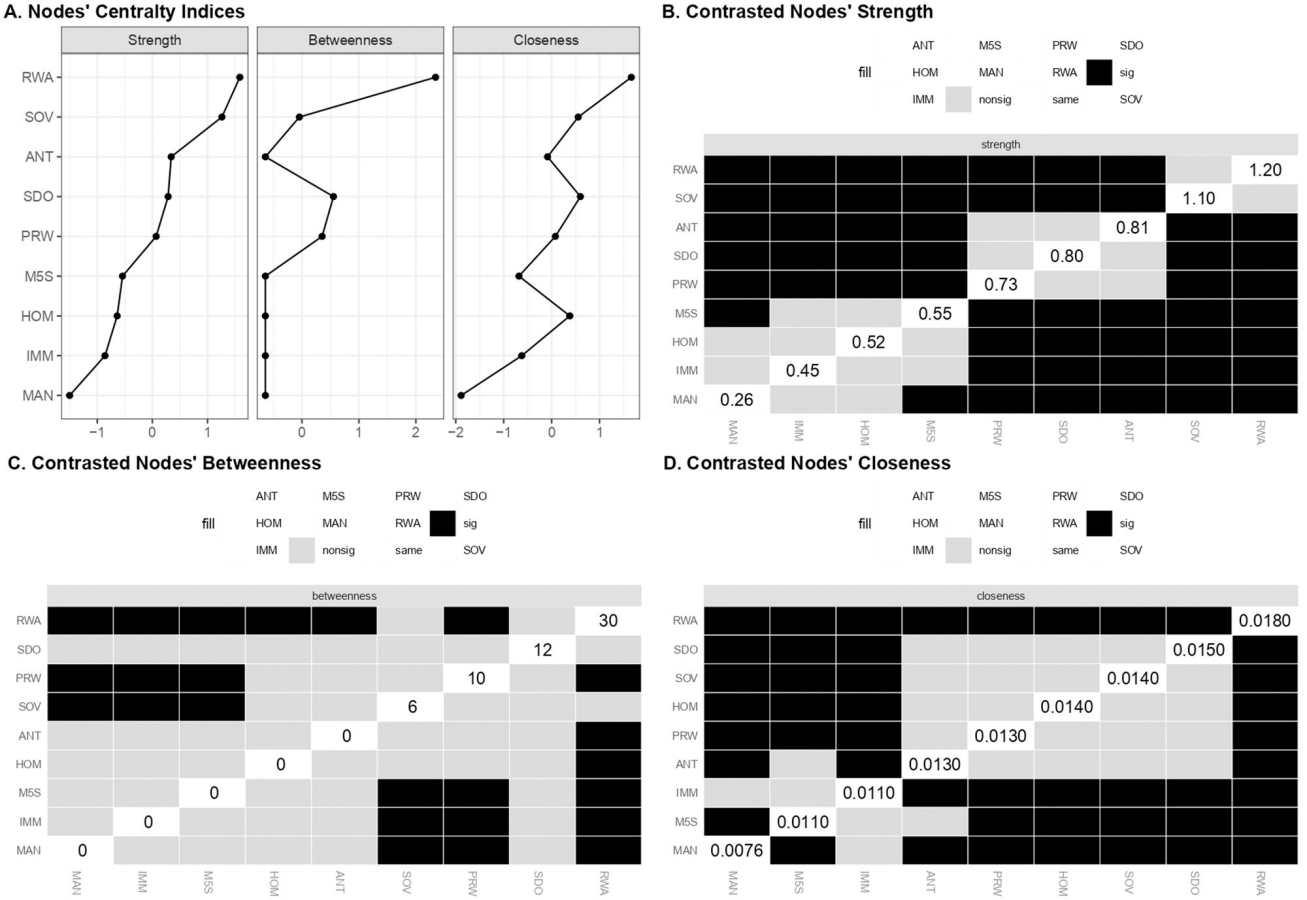
Estimates and bootstrap differences of centrality indices of the integrated beliefs system network. (A) Standardized values of centrality indices for each node of the network. (B) Bootstrap differences between nodes for the strength centrality index. (C) Bootstrap differences between nodes for the betweenness centrality index. (D) Bootstrap differences between nodes for the closeness centrality index. Fig 10B-10D report sample estimates on the diagonal, while bootstrap differences in the black and gray boxes. Black boxes indicate significant differences based on bootstrap mean estimates.

To summarize, RWA and SDO could be described as the core nodes of the network, since they were able to channel the associations among the other nodes. This cornerstone role transpired in their centrality indices (Fig 10A) and the related bootstrapped parameters (Table 8). RWA and SDO highlighted the highest value of closeness (Fig 10D) and betweenness (Fig 10C), with RWA prevailing significantly over all other nodes. Both ideological attitudes, and RWA in particular, transpired as being the core of the network. RWA’s central role was also indexed by its strength estimate (Fig 10B). Both socio-political dispositions were suitable for establishing connections with other pairs of nodes.

**Table 8:** Sample and bootstrap mean of centrality indices of the integrated beliefs system network including ideological attitudes, anti-immigration attitudes, populist ideology and voting.

	Strength					Betweenness					Closeness				
	*Estimates*			*95%CI*		*Estimates*			*95%CI*		*Estimates*			*95%CI*	
Node	*Sample*	*Mean*	*SD*	*Low*.	*Up*.	*Sample*	*Mean*	*SD*	*Low*.	*Up*.	*Sample*	*Mean*	*SD*	*Low*.	*Up*.
ANT	0.814	0.891	0.121	0.571	1.056	0	2.851	3.505	-7.010	7.010	0.013	0.014	0.001	0.010	0.016
HOM	0.519	0.592	0.107	0.306	0.733	0	2.493	3.553	-7.106	7.106	0.014	0.015	0.001	0.011	0.017
IMM	0.453	0.494	0.092	0.269	0.637	0	0.006	0.170	-0.339	0.339	0.011	0.012	0.001	0.009	0.013
M5S	0.548	0.630	0.122	0.304	0.793	0	0.868	1.355	-2.711	2.711	0.011	0.012	0.001	0.008	0.014
MAN	0.260	0.366	0.147	-0.035	0.555	0	0.000	0.028	-0.057	0.057	0.008	0.009	0.002	0.004	0.011
PRW	0.732	0.809	0.125	0.482	0.982	10	9.018	2.312	5.376	14.62	0.013	0.014	0.001	0.010	0.016
RWA	1.188	1.217	0.096	0.995	1.381	30	21.54	5.985	18.03	41.97	0.018	0.018	0.002	0.014	0.021
SDO	0.797	0.870	0.131	0.535	1.059	12	7.672	4.802	2.395	21.61	0.015	0.016	0.002	0.011	0.018
SOV	1.090	1.147	0.107	0.877	1.304	6	9.003	4.526	-3.052	15.05	0.014	0.016	0.002	0.011	0.017

**Fig 11:**
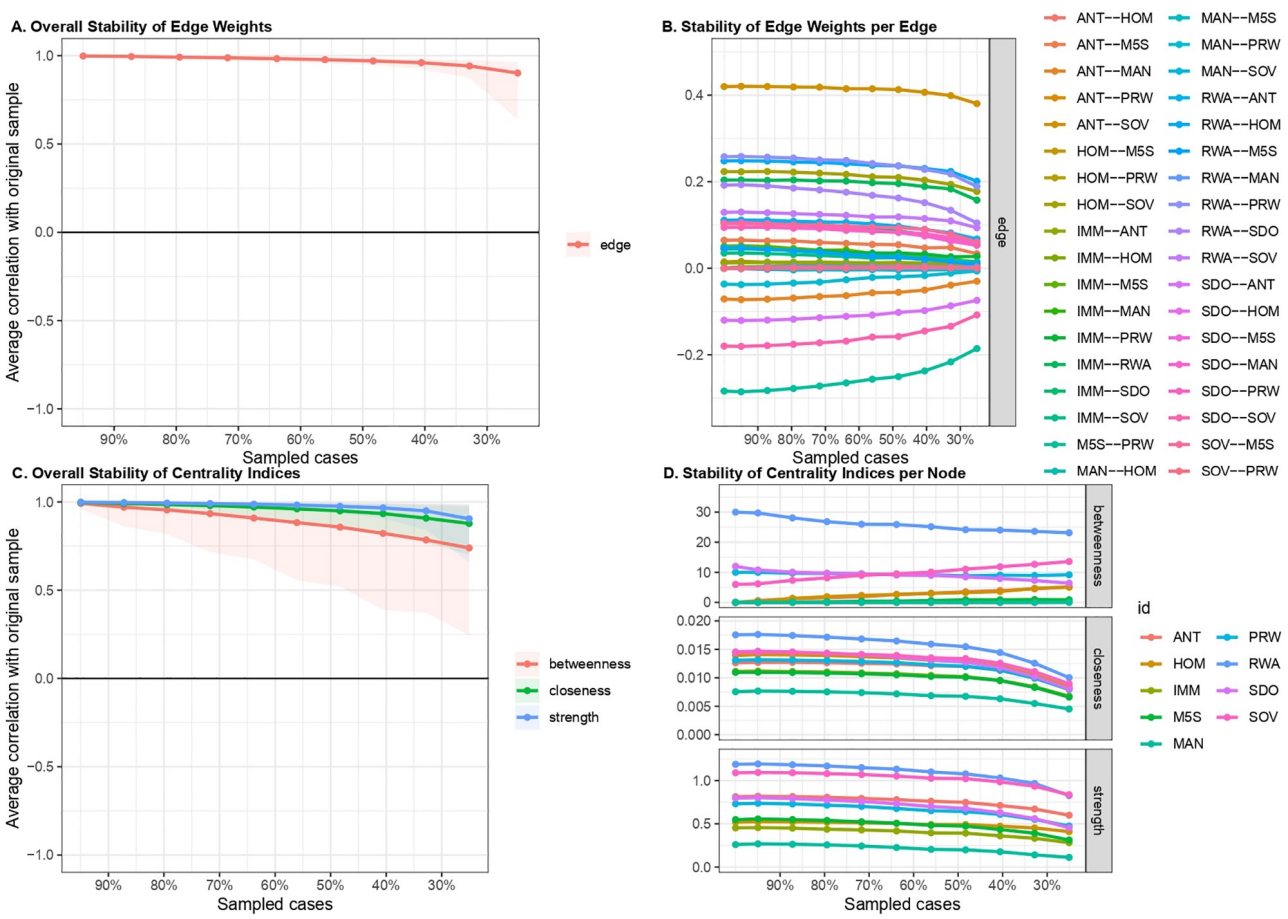
Stability of edge weights and of centrality indices of the integrated beliefs system network. (A) Overall stability of edge weights. (B) Stability of each edge weight. (C) Overall stability of estimated centrality indices. (D) Stability of estimated centrality indices for each node.

As for stability, the analysis revealed optimal parameters, with satisfactory stability even when a large portion of the sample was dropped. Estimated edges (Fig 11A and 11B), as well as centrality indices (Fig 11C and 11D), were stable until 75% of the sample was dropped. The *CS-coefficient* of the estimated edges (*CS*(*cor* = 0.7) = 0.75) and strength (*CS*(*cor* = 0.7) = 0.75) were highly satisfactory. Similarly, the estimates of closeness (*CS*(*cor* = 0.7) = 0.59) and betweenness (*CS*(*cor* = 0.7) = 0.36) were stable under subsetting cases. Hence, the network model and the related parameters were accurate.

## General discussion

The extensive research on populism has demonstrated remarkable convergence in the conceptual definition of the construct. By adopting the ideational approach [16], scholars agree in the description of populism as a thin-centered ideology (Mudde, 2004) clearly characterized by a multidimensional nature [e.g., 6, 7, 19]. Nevertheless, a slight incoherence has been manifested in establishing the content, the number, and the operationalization of its dimensions [17]. The present research thus examined the structure of populist ideology by exploring a combination of the main operationalizations that assess the ordinary citizens’ endorsement of such ideology. An explorative graph analysis highlighted the emergence of four potential constitutive dimensions which can be summarized as People Sovereignty, Anti-Elitism, People Homogeneity, and Manichaeism. Through the implementation of an innovative methodology, the results of the model merge its manifold operationalizations and converge with the conceptual features used to describe populism as a multidimensional thin-centered ideology. Although in purely descriptive terms, findings provide a more comprehensive picture that corroborates the conclusions of previous research focused on the operationalization of populist concept. However, the presence of multiple items that initially converged into different dimensions, or the small number of items for some of them, testifies to the need for a conceptual and methodological refinement of the construct’s operationalization strategies. For instance, the operationalization of the Manichean dimension may lack in semantic terms and in the definition of the actors engaged in the moral opposition between good and evil. The measures employed in the present research seem to focus on a moral polarization between ordinary people with divergent political ideas. However, the literature has sometimes described this distinction as a struggle between the “evil” elite and the “good” people [16] or, at times, as a general view of the functioning of society based on the two poles [57]. Shedding light on this aspect may represent a profitable aim for future research.

Scholars also underlined a persistent uncertainty about the conceptual core of populism [e.g., 17]. Thus, the present research provided a description of the main contents of populist ideology, the central beliefs that characterize it, and which dimensions are placed at its core. Despite extensive investigations of populist ideology and its characteristics, a description in terms of a network of interrelated beliefs and the centrality of each belief in the network has not been provided by the extant literature. Network analysis highlighted the dimensions of People Sovereignty as the core of populist ideology. The analysis also suggested that the dimensions of Anti-elitism and People Homogeneity cannot be overlooked, since they emerged as an integral part of the ideological network space of populism. Manichaeism was instead found as less integrated with the other constitutive dimensions and thus more peripheral. Individual adherence to populist ideology appeared to be mainly animated by a fervent desire to place power in the hands of the “true People” (People Sovereignty), which is perceived as an honest and virtuous entity (People Homogeneity), for opposing the crisis of criteria based on which the holders of power justify its exercise (Anti-elitism). In other words, the urgency to face a sentiment of alienation from the political reality fed by the idea that an unrepresentative powerful clique illegitimately wields political power for its own advantage. The view of the people as sharply polarized on moral grounds (Manichaeanism) emerged as a narrative that might be not shared transversally by all the individuals who adhere to populism. It represented a feature that might characterize only specific populist constituencies or, perhaps, a wider political audience that is not necessarily populist. This finding would corroborate the remark that the effort made in defining populism’s characteristics has sometimes encountered a conceptual overlap with valid public opinion constructs, thus undermining the conceptual meaning of populist attitudes [17]. However, the consideration of a refined Manichean outlook in future examinations of populism might also allow for identification of the peculiarities of its left-to-right or valence variants. Thus, the descriptive endeavor to trace a direction for orienting the efforts in populism’s investigation made herein may provide new insights for future research.

Furthermore, the present research also described how populist beliefs may be interrelated in a network with traditional socio-political attitudes (SDO and RWA). The network may be interpreted as a representation of the ideological opportunism and plasticity of populism, and of its ability to “feed” on more traditional ideologies. The provided description advances and corroborates literature that, having first prioritized the association of populism with macro-level factors [e.g., 33, 58, 59], has then turned to place emphasis on the psychological dynamics underlying populism [e.g., -10–12, 60–63].

Consistent with the present research’s expectations, right-wing authoritarianism and social dominance orientation were found to be essential in shaping the structure of the network model. Both ideological attitudes were central within the beliefs system and played a bridging role in connecting all the other variables. Focusing on the relationships of ideological attitudes with populist ideology’s dimensions, the results are consistent with the findings of Jost and Vasilopoulos [12]. Correlations and the network analyses empirically supported the idea that RWA and SDO relate to populism, each in its own peculiar fashion. RWA related positively to the pivotal populist dimensions of Anti-elitism, People Sovereignty, and People Homogeneity. RWA showed instead to be unrelated to Manichaeism. Thus, resistance-to-change motives appeared as a potential enabler of the endorsement of populism. Motivations to prevent reality from losing its meaning through an emphasis on traditional values and way of life appeared consistent with the tendency to delegitimize the “elite” perceived as intent on overthrowing the established social system at the expense of ordinary citizens. It is not surprising that these basic motivations were also consistent with the request to entrust power in the hands of a homogeneously virtuous entity (i.e., ordinary people) holding the steady ways of life and shared values that are threatened by the “evil” elite. Hence, the likely incompatibility of RWA with the Manichean polarization of the people. On the other hand, SDO resulted as negatively related to Anti-elitism and People Sovereignty, unrelated to People Homogeneity, and positively associated with Manichaeism. The inherent SDO’s proclivity to maintain order and stability through hierarchy-enhancing interactions among social groups emerges as the opposite with the desire to entrust the management of society to the popular will. Entrusting power into the hands of the people implies the flattening of hierarchies which are instead crucial for SDO. Similarly, SDO views the presence of leading social groups as an indispensable element for the proper functioning of society. This proclivity is the opposite of the anti-elitist tendency to reject and delegitimize the ruling class. Moreover, the SDO’s propensity to foster a clear and narrowed distinction between social groups on hierarchical power grounds is incompatible with a perception of the people as undifferentiated or homogeneous. People with a high SDO may hardly see the whole population as a homogeneous entity, suggesting that the notion of the “pure” people could be targeted to a more circumscribed group of individuals. For instance, individuals belonging to the same electorate (i.e., people who think like me politically). Thus, the same tendency to emphasize differences between social groups may be seen as easily superimposable to a Manichaean stark opposition among the abovementioned narrowed “pure people” and other entities (e.g., individuals, parties, institutions) with a different political idea.

Overall, RWA exceeded SDO when connected with the operationalized conceptual features of populist ideology, emerging as compatible with the pivotal dimensions of Anti-elitism, People Sovereignty, and Homogeneity of the People. Speculatively, it may be argued that the restructuring of the contemporary political system wished by populist voters might coincide with the demand for a reactionary change that re-establishes the “true” traditional popular values. This would be consistent with previous research which found populism to be associated with forms of collective nostalgia [e.g., 64]. The willingness to follow a strong and charismatic political leader, and Rousseau’s notion of the “Volonté Générale” [5] would not be so distinct after all. Instead, the pattern of associations pertaining SDO is consistent with the intuition that a hierarchical attitude may be in opposition to the tenets of populism that claim to directly descend from egalitarian political sources—namely Anti-elitism and People sovereignty. These findings are particularly interesting because, although conceptually distinct [13], RWA and SDO have been found positively correlated to each other, and both are positively associated with traditional conservative ideology [27]. The peculiar relationships shown by SDO and RWA with populism’s dimensions could suggest the need for a more in-depth understanding of the variants of the populist ideology’s support, and that populism’s operationalizations may be refined in order to capture and distinguish them.

This notion is further corroborated by the present study if we focus on the transition from “thin-to-thick” ideology that populism exhibits when it veers strongly to the right compared to the valence populist counterpart. Within the network model, voting for the two distinct variants of populist parties (i.e., right-wing vs. valence) showed opposite patterns of associations with ideological attitudes and populist dimensions. For PRW (vs. M5S), RWA and SDO are seen to perform similar roles. PRW voting was found to be directly associated with SDO and RWA but unrelated directly to any of the populist ideology dimensions. In contrast, the vote for the M5S showed robust direct associations with the dimensions of Anti-elitism and People Sovereignty, but no direct association with both ideological attitudes. These results instantiated an interesting polarization of ideological and populist attitudes across distinct variants of populist supporters. Support for conservative parties is usually motivated by the emphasis on maintaining the status quo through authoritarianism and social dominance [e.g., 27]. In the present study, PRW voting emerged animated by the same basic motivations and, importantly, by none of the dimensions considered as the content of populist ideology. Moreover, classical right-wing voters are known to manifest prejudicial attitudes towards minority groups such as immigrants [e.g., 65, 66]. Consistently, in the network model, PRW voters resulted indirectly close to the adoption of anti-immigrant stances. The association between right-wing populist voting and anti-immigration attitudes was uniquely channeled by both SDO and RWA. Right-wing populism appeared close to traditional forms of right-wing ideology support. In contrast, vote for the M5S was directly connected with Anti-elitism and People Sovereignty, thus presenting a close connection with two essential contents of populist ideology. Support for the valence populism variant was distant from the respective right-wing form of populist support. M5S voting was not in direct relation to both ideological attitudes. It related indirectly solely to RWA, however passing through populist dimensions. This association pattern also placed the M5S at lesser indirect proximity to anti-immigration attitudes. It appears that the M5S may represent an example of a purer form of populism, as compared with the right-wing variant. Consistent with the notion of valence populism of Zulianello [21], M5S appeared as firstly animated by non-ideological issues or, rather, by those that could be the contents of a non-traditional ideology, such as populism claims to be. Classical ideology contents were secondary to populist ideology for M5S voters. They emerged as a probable consequence of the contradictor and opportunistic oscillation of valence populism across different tenets, without becoming entangled in a fixed position on the left–right spectrum.

Wuttke et al. [17] argued that the implementation of continuous measures for the assessment of populism is likely to be lacking in successfully capturing its political manifestations among different groups of voters. This limit can be overcome by adopting a non-compensatory view of populism as an attitudinal syndrome: a construct that needs the simultaneous presence of its distinctive features to be encountered. Otherwise, categorical measures that enable for clustering of populist voters could be adopted [17]. Working in this direction, the present research has considered the self-reported vote as an operational strategy for classifying populist voters, as well as their related variants. Voting is herein intended as part of a dynamic system where different elements may exert reciprocal influence [67]. The implementation of a network model allowed for the examination of the viability of viewing populism as a non-compensatory attitudinal syndrome by investigating the simultaneous presence of weighted associations between populist dimensions, specific clusters of populist voters, and other traditional ideological constructs. The whole associative pattern suggests that in order to adopt a non-compensatory approach to populism, an accurate and robust preliminary definition of its constitutive dimensions is mandatory. Moreover, the lack of direct association between PRW and populist dimensions draws attention to the potential risks of considering populism as an attitudinal syndrome. In particular, it highlights the possibility of ignoring populism when it is embodied in its most extreme form. At the same time, the association patterns for M5S voters suggest that the dimensions of populism may not occur simultaneously to explain support for a party. The M5S can be considered a meaningful example of what populist ideology aims to be in its essence. That is, a political reality free from classic ideological connotations that bases its roots on themes such as fight against corruption, greater transparency, democratic reform, and moral integrity.

This reasoning paves the way for another interesting theoretical speculation. Populism is often described as subordinate to its host ideologies. That is, a set of thin ideologies that can be adopted as rhetorical and political strategies to make the primary ideology more appealing to certain audiences [e.g., 68]. The findings relating to the PRW that emerge in the present study seem to corroborate this view. However, if we focus on an apparently purer form of populist ideology (i.e., valence populism), the findings pertaining to M5S may suggest a potential reversal pattern. That is, the ability of a potential non-traditional ideology to use the ideological connotations of classical ideologies to opportunistically gain consensus. Future research may disentangle such an interesting interrogative.

## Limitations and conclusions

Some limitations need to be acknowledged. The present study is based on data collected in Italy through a snowball sampling procedure, which might limit the generalization of results. However, findings are consistent with previous research focusing on other different national contexts and involving representative sample [e.g., 10, 12, 63, 69], which may attest to a fair degree of conceptual generalizability of my conclusions. Moreover, data have been collected in a period after populist parties obtained a major electoral victory but before they formed a government. Recent research [e.g., 57, 70] show that consensus for populists can change rapidly once they are in the government. Therefore, future investigations focused on this specific side of populist support are needed to corroborate the findings of the present research. The explicit descriptive approach of the present research may be read as a further limitation. Notwithstanding, I believe the description is not a limitation, but a necessary preliminary step, when the profiles of phenomena are still unclear, relatively unknown in their complete development, and indeed “fuzzy.” The descriptive approach could be also seen as limiting the retrieval of robust inferences from the tested associations. However, the present study does not claim to establish causal links between the variables examined. Rather, it aims to provide descriptive cues that may be the object of future investigations aimed at establishing causation. The implementation of partial correlations network analysis seems to be a particularly appropriate strategy for pursuing this aim since they provide highly exploratory hypothesis-generating structures indicative of potential causal effects [43]. Another limitation might be the unsatisfactory reliability score obtained for People-centrism and the Manichean Outlook subscales of Castanho Silva et al.’s [7] measure. However, this issue was efficiently dealt with by the exploratory graph analysis which provided an accurate definition of populist dimensions, preventing potential repercussions in the network models where variables were treated at aggregated scale-level.

To conclude, regardless of the variation between different strains of populism, RWA and SDO represented psychological dimensions from which populism may draw defined policy- and ideology-rich content. The findings about the pivotal role of individual differences pertaining to hierarchy-enhancing and resistance-to-change motives imply the presence of deep psychological concerns among populist voters that should not be underestimated by political entities alternatives to populism. The neglect and stigmatization of this section of the electorate have created fertile ground for the growth of consensus in favor of populist leaders and parties that fueled a xenophobic and exclusionist view of society. In the United States presidential election of 2016, the democratic candidate Hillary Clinton dealt a severe blow to the success of her electoral campaign by using the appellative “basket of deplorables” toward Trump supporters. Many Trump supporters adopted such an appellative for themselves, eliciting a process of semantic change of the term which, from derogatory, has come to represent a reason for pride and distinction. Rather than stigmatizing populism and its electorate, the focus could be shifted to reducing voters’ sentiments of alienation from the political reality and laying solid foundations for a more equitable and fair future, possibly perceived as less threatening and frightening.

## Supporting information

S1 File Partial correlation matrices of EGA model of the initial and final network structure of populist ideology. (XLSX)

S2 File Dataset. (SAV)
